# Differential expression profile of CXCR3 splicing variants is associated with thyroid neoplasia. Potential role in papillary thyroid carcinoma oncogenesis?

**DOI:** 10.18632/oncotarget.23502

**Published:** 2017-12-20

**Authors:** Soledad Urra, Martin C. Fischer, José R. Martínez, Loreto Véliz, Paulina Orellana, Antonieta Solar, Karen Bohmwald, Alexis Kalergis, Claudia Riedel, Alejandro H. Corvalán, Juan C. Roa, Rodrigo Fuentealba, C. Joaquin Cáceres, Marcelo López-Lastra, Augusto León, Nicolás Droppelmann, Hernán E. González

**Affiliations:** ^1^ Department of Surgical Oncology, Faculty of Medicine, Pontificia Universidad Católica de Chile, Santiago, Chile; ^2^ Department of Physiology, Faculty of Biological Sciences, Pontificia Universidad Católica de Chile, Santiago, Chile; ^3^ Department of Pathology, Faculty of Medicine, Pontificia Universidad Católica de Chile, Santiago, Chile; ^4^ Millennium Institute on Immunology and Immunotherapy, Department of Molecular Genetics and Microbiology, Faculty of Biological Science, Pontificia Universidad Católica de Chile, Santiago, Chile; ^5^ Millennium Institute on Immunology and Immunotherapy, Department of Endocrinology, Faculty of Medicine, Pontificia Universidad Católica de Chile, Santiago, Chile; ^6^ Millennium Institute of Immunology and Immunotherapy, Department of Cell Biology, Faculty of Biological Science and Faculty of Medicine, Universidad Andrés Bello, Santiago, Chile; ^7^ Advanced Center for Chronic Diseases (ACCDiS), Department of Hematology and Oncology, Faculty of Medicine, Pontificia Universidad Católica de Chile, Santiago, Chile; ^8^ Institute of Biomedical Sciences, Faculty of Health Sciences, Universidad Autónoma de Chile, Santiago, Chile; ^9^ Laboratory of Molecular Virology, Millennium Institute of Immunology and Immunotherapy, Department of Infectious Diseases and Pediatric Immunology, School of Medicine, Faculty of Medicine, Pontificia Universidad Católica de Chile, Santiago, Chile

**Keywords:** CXCR3, CXCL10, papillary thyroid cancer, inflammation, chemokine receptors

## Abstract

Papillary thyroid cancer (PTC) is the most prevalent endocrine neoplasia. The increased incidence of PTC in patients with thyroiditis and the frequent immune infiltrate found in PTC suggest that inflammation might be a risk factor for PTC development. The CXCR3-ligand system is involved in thyroid inflammation and CXCR3 has been found upregulated in many tumors, suggesting its pro-tumorigenic role under the inflammatory microenvironment. CXCR3 ligands (CXCL4, CXCL9, CXCL10 and CXCL11) trigger antagonistic responses partly due to the presence of two splice variants, CXCR3A and CXCR3B. Whereas CXCR3A promotes cell proliferation, CXCR3B induces apoptosis. However, the relation between CXCR3 variant expression with chronic inflammation and PTC development remains unknown. Here, we characterized the expression pattern of CXCR3 variants and their ligands in benign tumors and PTC. We found that CXCR3A and CXCL10 mRNA levels were increased in non-metastatic PTC when compared to non-neoplastic tissue. This increment was also observed in a PTC epithelial cell line (TPC-1). Although elevated protein levels of both isoforms were detected in benign and malignant tumors, the CXCR3A expression remained greater than CXCR3B and promoted proliferation in Nthy-ori-3-1 cells. In non-metastatic PTC, inflammation was conditioning for the CXCR3 ligands increased availability. Consistently, CXCL10 was strongly induced by interferon gamma in normal and tumor thyrocytes.

Our results suggest that persistent inflammation upregulates CXCL10 expression favoring tumor development via enhanced CXCR3A-CXCL10 signaling. These findings may help to further understand the contribution of inflammation as a risk factor in PTC development and set the basis for potential therapeutic studies.

## INTRODUCTION

Thyroid cancer has one of the fastest growths in incidence when compared to other malignant and endocrine neoplasia [1, 2], where papillary thyroid cancer (PTC) represents approximately 80% of thyroid malignancies [3, 4]. Based on recent data showing a sharp increase in its rate of incidence, PTC is predicted to become the fourth most common malignancy by 2030 in United States [5]. Usually, 20% of patients develop lymph node metastasis (LNM) which is associated with increased patient morbidity and mortality [6, 7].

A remarkable progress has been made in the understanding of the oncogenic mechanisms that promote thyroid cancer development and progression. The inflammatory state of premalignant and malignant lesions play decisive roles in tumor initiation and progression [8]. Specific chronic inflammatory conditions that increase the risk of cancer development by promoting the infiltration of inflammatory cells to premalignant lesions and the oncogene signaling that initiate cancer by inducing the inflammation-related genes expression are known mechanisms that link inflammation with cancer [9]. Chronic thyroiditis has been reported in 20 to 50% of PTC cases [10, 11] and is associated to an increased risk of developing PTC [12–14]. Also, the higher incidence of PTC in patients with chronic lymphocytic thyroiditis or Hashimoto autoimmune thyroiditis (HT) [15–17] has suggested a role of inflammation as a predisposing factor in the development of thyroid cancer [18–20]. Even in the absence of any signs of HT, remarkable peritumoral lymphocytic infiltrate or focal lymphocytic thyroiditis is frequently found in PTC [18] which is significantly higher and more frequent than in benign thyroid lesions [21]. As lymphocytic infiltrate is generally found in the peritumoral environment of PTC, it has been suggested that it favors the development of PTC [22]. Thyroid cancers arising in the context of HT exhibit a better prognosis than PTCs without chronic lymphocytic thyroiditis [23, 24]. In the absence of typical signs of HT, poorly differentiated and anaplastic thyroid carcinomas, which are associated with a worse prognosis, generally exhibit a reduced lymphocytic infiltrate compared to PTCs [11]. However, there is still no clear evidence whether thyroid inflammation promotes tumor development and/or plays a protective role against thyroid cancer progression.

Phenomena related with inflammation, such as elevated cytokine and chemokine levels, have been suggested to predispose to RET proto-oncogene rearrangements (RET/PTC), a classic oncogene that induced malignant transformation in thyrocytes [18]. In turn, RET/PTC promoted PTC oncogenesis through a transcriptional program that included upregulation of chemokines and their receptors creating an inflammatory environment that triggers a pro-tumorigenic response [18, 25–29]. Furthermore, increased levels of proteins from this “inflammatory program” have been detected in primary thyroid tumors of patients with LNM [25, 30]. Therefore, accumulating evidence suggests that there is a strong association between inflammation and increased risk to neoplastic transformation and progression in thyroid cancer [18–20, 31–33].

Inflammation is a key component of the tumor microenvironment and chemokines are part of the inflammatory network of mediators associated to neoplasia [34]. Frequently, chemokines and chemokine receptors are found in tumors and often their expression and signaling are deregulated [35, 36]. In fact, chemokine receptor signaling in malignant cells promotes tumor growth, invasion and metastasis [37–41]. Spread of tumor cells to chemokine gradients is restricted to specific patterns of chemokine receptors and to chemokine availability in the tumor microenvironment [42, 43]. Inflammatory cytokines produced by tumor cells and/or by tumor associated leukocytes may contribute to malignant progression [44]. Growing amount of evidence indicate that IFN-ɣ inducible chemokines (CXCL9, CXCL10 and CXCL11) and CXCR3 (their main receptor) [45, 46] play an important role in the initial stage of autoimmune thyroiditis [47, 48].

In thyroid tissues, secreted levels of CXCL10 are associated with T helper 1 (Th-1) cell infiltration which is commonly found in autoimmune thyroiditis and has been closely related to thyroid tumors [49]. Th-1 lymphocytes recruited to a tumor site may be responsible for enhanced production of IFN-γ and tumor necrosis factor-α (TNF-α) which in turn stimulates CXCL10 secretion from a variety of cells helping to maintain and potentiate cytokine production in the tumor microenvironment [50]. Since IFN-γ induces the secretion of CXCR3 ligands and CXCL10 in normal [51, 52] and PTC thyrocytes [53], it has been proposed that CXCR3 chemokines may promote thyroid malignant transformation and/or thyroid cancer progression [54–57]. In fact, the CXCR3 system plays a pivotal role in the malignancy process. The CXCR3 angiostatic activity is complemented with its ability to potentiate the anti-tumor immunity in several tumor systems [58–60]. However, upregulation of the receptor has been reported in many human tumors and is frequently associated with LNM and poor prognosis [61]. IFN-γ inducible chemokines have shown to promote malignancy by inducing cell migration of solid tumors to metastatic sites [62–64]. In lymph nodes, CXCL9 and CXCL10 upregulation is induced by activated Th-1 lymphocytes that secretes IFN-γ [62, 65, 66]. Moreover, CXCR3 expression levels in primary tumor were causatively involved in LNM [62]. These observations suggest that host and tumor cells can exploit IFN-γ inducible chemokines to promote their neoplastic transformation and cancer progression through CXCR3 signaling.

CXCR3 chemokines may have divergent effects on tumor cells in part dictated by the array of two functionally CXCR3 splicing variants; CXCR3A and CXCR3B [46, 67]. While CXCR3A signaling (by CXCL10 and CXCL11) promotes cell viability, proliferation, angiogenesis and inhibits apoptosis, CXCR3B promotes an angiostatic effect and apoptosis [46]. The relative expression of CXCR3 variants is important for regulating proliferation and survival in several cancer cells [67–69] and correlates with tumor dissemination and metastasis [70–73]. In human renal, ovarian, prostate and breast cancer tissues, CXCR3A is upregulated while CXCR3B is markedly decreased [67, 68, 73, 74]. Although mRNA levels for CXCR3, CXCL10 and CXCL11 were increased in thyroid cells carrying mutations in the RET/PTC-RAS-BRAF axis [27], no studies have addressed the contribution for the CXCR3-ligand system in PTC development and LNM.

In this study, we determined the expression pattern of CXCR3 variants and their ligands in PTC and in benign thyroid tumors and compared them with non-malignant thyroid tissue. In addition, to investigate if the isoform expression profile is associated with cancer progression or inflammation, we analyzed this pattern in patients with and without LNM or chronic thyroiditis. Here we demonstrate, to our knowledge, for the first time that protein levels of both CXCR3 isoforms are progressively upregulated in benign and malignant tumors where CXCR3A expression remains as the predominant isoform and promotes cell proliferation in Nthy-ori-3-1 by CXCL10 and CXCL11 stimulation. CXCL10 is upregulated only in PTC patients with thyroiditis and is significantly induced by IFN-γ in normal and cancer epithelial cells. Overall, our findings suggest a potential role of inflammation in PTC tumorigenesis through CXCR3A-CXCL10 enhanced proliferative signaling.

## RESULTS

### Increased CXCR3 expression in human PTC tissues

**Figure 1:**
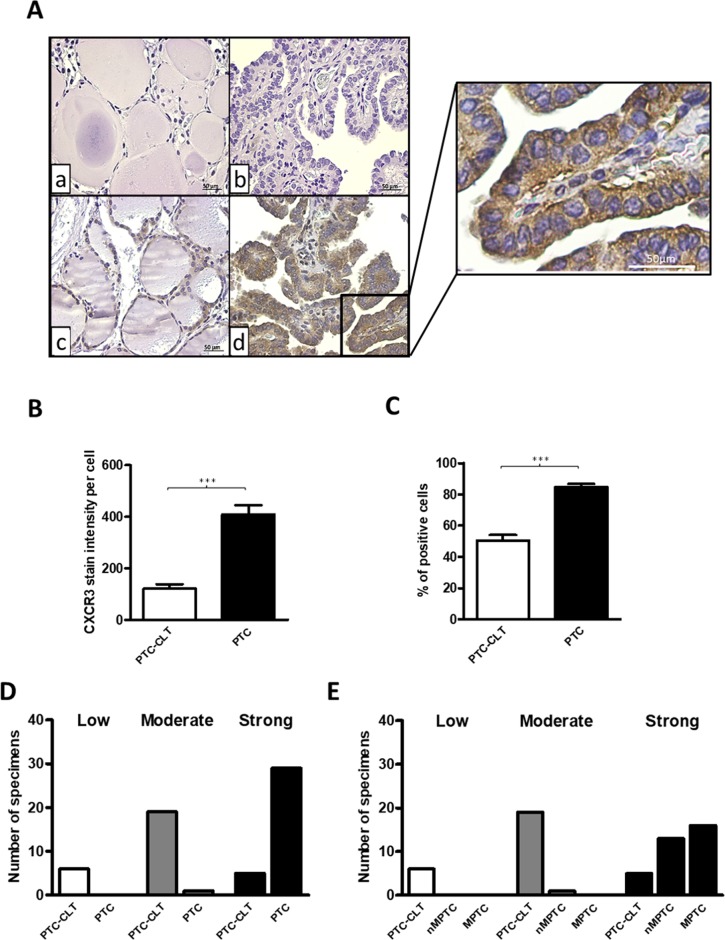
Increased CXCR3 expression in human PTC tissues **(A)** Representative photomicrographs from immunohistochemistry analysis of CXCR3 expression in PTC tumors and their matched contralateral tissues (PTC-CLT). PTC-CLT (a, c) and PTC (b, d) tissue samples incubated with primary antibody (c, d) or left without CXCR3 antibody (a, b) for background determination. Inset = Higher magnification of the area in the frame d. Scale bar = 50 μm. Magnifications in 400x. **(B)** Quantitative analysis of CXCR3 staining intensity in 30 PTC tumors and their PTC-CLT was performed as described in materials and methods. **(C)** Quantitative analysis of percentage of positive cells for CXCR3 immunostaining in 30 PTC tumors. **(D)** Frequency graph of low, moderate and high percentage of positive cells for CXCR3 immunostaining in 30 PTC tumors and 30 PTC-CLT. 14 PTC specimens were non-metastatic PTC (nMPTC) and 16 metastatic PTC (MPTC) **(E)**. Data was plotted as bar graphs and represent mean ± s.e.m. Statistical analysis was performed using a paired two-tailed t-test (^***^p<0.001).

CXCR3 expression was analyzed by immunohistochemistry in 30 PTC tumors (14 non-metastatic PTC (nMPTC) and 16 metastatic PTC (MPTC) and in their contralateral tissue. Histological features for PTC specimens and distribution of PTC tumors analyzed by immunohistochemistry are shown in Table 1. CXCR3 was mainly detected in follicular cells (Figure 1A, section d and inset). The quantitative analysis of immunoreactivity showed a 4-fold increase of average staining intensity for CXCR3 expression in PTC (409.5±179.8) vs. PTC-CLT (121.2±89.3, p<0.0001) (Figure 1B). CXCR3 immunoreactivity showed reduced risk to develop extrathyroidal extension (OR 0.09, 95% IC 0.01–0.74) (Table 2). No associations were found between CXCR3 staining with the others histopathological features evaluated (tumor size, lymph vascular invasion, LNM and thyroiditis). Additionally, the percentage of positive cells for CXCR3 immunostaining was higher in PTC (85%) than in PTC-CLT (51%, p<0.0001) (Figure 1C). The frequency graph of CXCR3 positive cells showed that most of PTC specimens presented high frequencies (% of CXCR3 immune reactive cells between 67-100%), whereas PTC-CLT cases were mainly distributed in moderate (34-66%) and low frequencies (0-33%) (Figure 1D). A representative image of low, moderate and high percentage of positive CXCR3 reactive cells is shown in Supplementary Figure 1. When the CXCR3 frequency of positive cells was analyzed between MPTC and nMPTC specimens, no differences were found (Figure 1E). Thyroiditis was found in 30% of PTC specimens and all cases with chronic inflammation showed a high frequency for CXCR3 reactive cells. No differences were found in CXCR3 staining intensity between cases with or without thyroiditis. Moreover, PTC-CLT with or without thyroiditis, showed similar CXCR3 intensity levels (Supplementary Figure 2).

**Table 1:** Histopatological features of PTC specimens analyzed by immunohistochemistry

	PTC Specimens (n=34)
**Sex**	
Male	8 (24%)
Female	26 (76%)
**Age (years)**	
< 45	18 (53%)
≥ 45	16 (47%)
**Size (cm)**	
< 2	13 (38%)
> 2	21 (62%)
**Histology (Cancer)**	
PTC - UV	24 (71%)
PTC - FV	10 (29%)
**Lymphonodal Metastasis**	
No	18 (53%)
Central	5 (15%)
Lateral	11 (32%)
**Thyroiditis**	
No	22 (65%)
Yes	12 (35%)

For immunohistochemistry analysis, the distribution of PTC tumors was, PTC - UV (Usual variant, 24 cases), PTC - FV (Follicular variant, 10 cases). PTC with LNM (MPTC, 16 cases) and PTC patients without LNM (nMPTC, 18 cases). PTC specimens with thyroiditis were 12 cases and without thyroiditis, 22 cases.

**Table 2:** Association between histopathological features and CXCR3 immunoreactivity by univariate analysis

	Tumor Size (≥ 2 cm)	Extrathyroid Extension	Lymphovascular Invasion	Lymphonodal Metastasis	Thyroiditis
**Immunoreactivity**					
Odds Ratio	1.38	**0.08**	1	0.727	2.56
95% CI	0.29 - 6.60	**0.01 - 0.74**	0.19 - 5.24	0.15 - 3.49	0.49 - 13.39

Significative results are showed in bold font.

### CXCR3A and CXCL10 mRNA levels increase in non-metastatic PTC tissues

**Figure 2:**
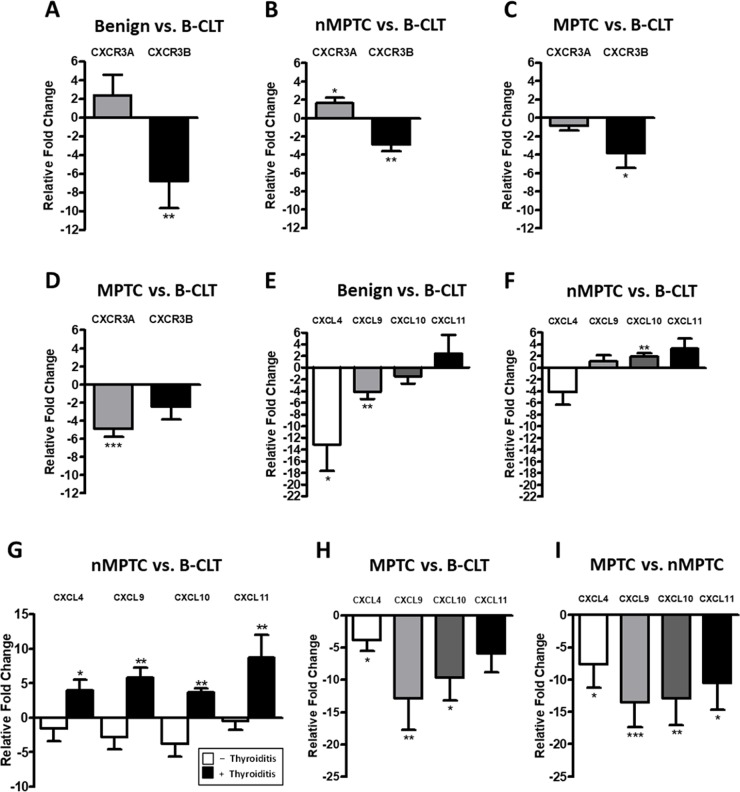
CXCR3A and CXCL10 mRNA levels increases in non-metastatic PTC tissues mRNA expression levels of target genes were determined by qPCR in benign and PTC tumors and in their respective matched contralateral tissues. Ct values were plotted using the Pfaffl's mathematical model. **(A)** Relative fold change (RFC) of CXCR3A and CXCR3B in 30 benign tumors **(B)** in 40 non-metastatic PTC (nMPTC) and **(C)** in 35 metastatic PTC (MPTC) compared to 30 benign contralateral tissues (B-CLT). **(D)** RFC of CXCR3A and CXCR3B in 35 MPTC compared to 40 nMPTC. **(E)** RFC for CXCL4 (n=17), CXCL9, CXCL10 and CXCL11 (n=30) in benign tumors compared to 30 B-CLT. **(F)** RFC for CXCL4 (n=28), CXCL9, CXCL10 and CXCL11 (n=40) in nMPTC compared to 30 B-CLT. **(G)** RFC for CXCL4 (n=16 without thyroiditis, n=12 with thyroiditis), CXCL9, CXCL10 and CXCL11 (n=26 without thyroiditis, n=13 with thyroiditis) in nMPTC compared to 30 B-CLT. **(H)** RFC of CXCL4 (n=21), CXCL9, CXCL10 and CXCL11 (n=34) in MPTC compared to 30 B-CLT. **(I)** RFC of CXCL4 (n=28), CXCL9, CXCL10 and CXCL11 (n=35) in MPTC compared to 40 nMPTC. In all graphs, data is shown as mean ± s.e.m. Statistical significance for Pfaffl's analyses was performed using the Wilcoxon test (^*^p<0.05, ^**^p<0.01, ^***^p<0.001).

For qPCR and western blot analyses the histological features for benign and PTC specimens and their corresponding distribution is shown in Table 3. Primer sequences for CXCR3 variants and their ligands are listed in Table 4. Next we analyzed the mRNA levels for CXCR3A and CXCR3B in benign nodules and in their contralateral tissue (B-CLT) (control tissue). CXCR3A was slightly but not significantly increased and CXCR3B was reduced by 7-fold compared to B-CLT (-6.801±15.41, p=0.0073) (Figure 2A). Then, we investigated if changes in CXCR3 mRNA profile could be associated to tumor development by comparing the expression of CXCR3A and CXCR3B in nMPTC with B-CLT. Expression of CXCR3A was significantly increased (1.651±3.539, p=0.0091) whereas CXCR3B was decreased (-2.903±4.368, p=0.0011) (Figure 2B). This indicates that in PTC the expression profile of CXCR3 variants is represented by increased levels of the pro-tumoral CXCR3A together with a reduction of the anti-tumoral CXCR3B isoform. To determine if the profile exhibited by the CXCR3 variants is associated to tumor metastatic progression, mRNA levels of CXCR3 variants were compared between MPTC and B-CLT (Figure 2C) and between MPTC and nMPTC (Figure 2D). A significant decrease of CXCR3B expression was observed in MPTC (-3.84±8.66, p=0.0139) when compared to B-CLT, while CXCR3A levels remained constant (Figure 2C). In contrast, when mRNA levels were compared using nMPTC as control tissue, a significant decrease of CXCR3A expression was observed in MPTC (-4.876±5.279, p<0.0001) while CXCR3B did not significantly change (Figure 2D). To investigate the potential role of CXCR3 ligands in the process of neoplastic transformation and tumor progression, the mRNA levels of CXCR3 ligands were assessed in benign and PTC tissues. We analyzed ligands expression in benign nodules (B), nMPTC and MPTC by comparing to B-CLT. In benign nodules, CXCL11 was increased by two folds (2.401±17.29), although without statistical significance whereas CXCL4 (-13.13±18.74, p=0.0121) and CXCL9 (-4.132±6.374, p=0.0044) were decreased (Figure 2E). In addition, the analysis of CXCR3 ligands revealed that the expression of CXCL10 and CXCL11 were increased in nMPTC (1.883±2.950, p=0.0023; 3.307±9.782) while CXCL4 mRNA levels were decreased (-4.126±10.33) (Figure 2F). When comparing CXCR3 ligands expression in nMPTC with or without thyroid inflammation respect to B-CLT, a significant increase of all ligands only in nMPTC with thyroiditis (CXCL4: 3.236±3.855, p=0.469; CXCL9: 5.021±4.166, p=0.0039; CXCL10: 2.713±1.574, p=0.0039; CXCL11: 8.121±10.21, p=0.0098) was observed (Figure 2G). In contrast, all ligands were downregulated in nMPTC without thyroiditis. Comparison of CXCR3 ligands expression in MPTC respect to B-CLT showed that CXCL4, CXCL9 and CXCL10 were significantly reduced (Figure 2H). Similar results were obtained when nMPTC was used as a control tissue. All ligands were decreased with statistical significance in MPTC, with major decreases for CXCL9 (-12.86±21.07, p=0.0002), CXCL10 (-12.58±23.08, p=0.0015) and CXCL11 (-10.35±23.08, p=0.0171), all high affinity CXCR3A ligands [46]. Summarizing, the results indicate that CXCR3A is upregulated in nMPTC, while both CXCR3 variants and all their ligands are significantly decreased in MPTC. In nMPTC, the presence of thyroiditis conditioned the increased expression of all CXCR3 ligands, but CXCL10 levels increased significantly when compared to B-CLT.

**Table 3:** Histopathological features of PTC specimens analyzed by qPCR and western blot

	PTC specimens (n=85)	Benign specimens (n=44)
**Sex**		
Male	19 (22%)	9 (20%)
Female	66 (78%)	35 (80%)
**Age (years)**		
< 45	55 (65%)	15 (34%)
≥ 45	30 (35%)	29 (66%)
**Size (cm)**		
< 2	62 (73%)	21 (48%)
> 2	23 (27%)	23 (52%)
**Histology (Cancer)**		
PTC - UV	59 (69%)	
PTC - FV	14 (16%)	
PTC - HCV	6 (7%)	
PTC - TCV	2 (2%)	
PTC - DSV	4 (5%)	
**Histology (Benign)**		
Hyperplastic Nodules		30 (68%)
Follicular Adenomas		14 (32%)
**Multifocality**		
No	66 (78%)	
Yes	19 (22%)	
**Extrathyroid Extension**		
No	59 (69%)	
Yes	26 (31%)	
**Lymphovascular Invasion**		
No	74 (87%)	
Yes	11 (13%)	
**Lymphonodal Metastasis**		
No	36 (42%)	
Central	30 (35%)	
Lateral	19 (22%)	
**Thyroiditis**		
No	59 (69%)	32 (73%)
Yes	26 (31%)	12 (27%)

For qPCR and Western blot analysis the distribution of PTC tumors was, PTC - UV (Usual variant, 59 cases), PTC - FV (Follicular variant, 14 cases), PTC - HCV (Hurthle Cell variant, 6 cases), PTC - TCV (Tall Cell variant, 2 cases) and PTC-DSV (Diffuse-Sclerosant variant, 4 cases). Benign thyroid tumors included HP (Hyperplastic nodules, 30 cases) and FA (Follicular adenomas, 14 cases). PTC with LNM (MPTC) were 49 cases and PTC patients without LNM (nMPTC) were 36 cases. Benign specimens with thyroiditis were 12 cases and without thyroiditis were 32 cases. PTC specimens with thyroiditis were 26 cases and without thyroiditis were 59 cases.

**Table 4:** Primers

Gene	Sequence (5’ 3’)	Product (pb)
CXCR3	F: GTGGACATCCTCATGGACCT	70
	R: CGGAACTTGACCCCTACAAA	
CXCR3A	F: TGGTCCTTGAGGTGAGTGAC	190
	R: AAGAGGAGGCTGTAGAGGGC	
CXCR3B	F: AAGTACGGCCCTGGAAGACT	171
	R: GGCGTCATTTAGCACTTGGT	
CXCL4	F: TGAAGAATGGAAGGAAAATTTGC	91
	R: GCAGCTAGTAGCTAACTCTCCAAAAGT	
CXCL9	F: GTTCTGATTGGAGTGCAAGGA	146
	R: ATGATTTCAATTTTCTCGCAGG	
CXCL10	F: TTCAAGGAGTACCTCTCTCTAG	177
	R: CTGGATTCAGACATCTCTTCTC	
CXCL11	F: GTGAAGGGCATGGCTATAGC	161
	R: TTGTTACTTGGGTACATTATGGAGG	
RNA18S	F: CGGTACAGTGAAACTGCGAAT	154
	R: TCTGATAAATGCACGCATCC	
ACTB	F: CTCTTCCAGCCTTCCTTCCT	116
	R: AGCACTGTGTTGGCGTACAG	

### A progressive increase in protein balance of CXCR3A/CXCR3B from benign tumors to PTC tissues

**Figure 3:**
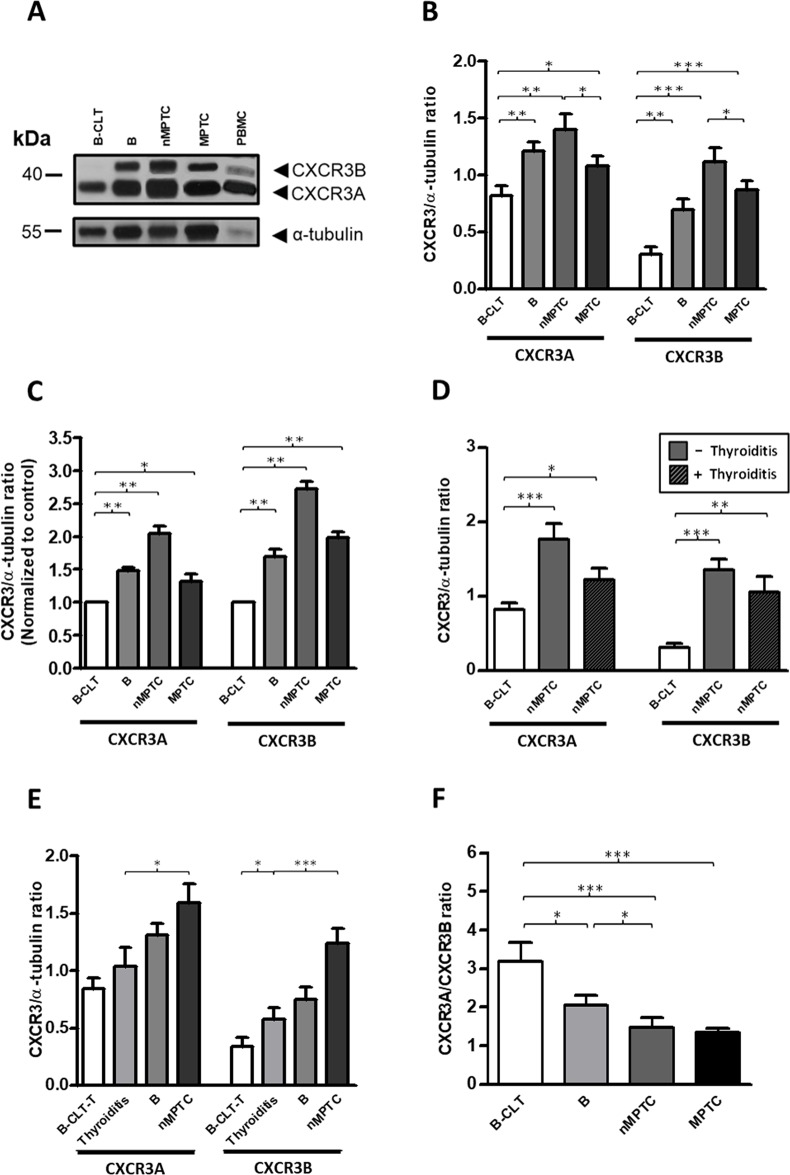
A progressive increase in protein balance (CXCR3A/CXCR3B ratio) from benign to PTC tissues Protein levels of CXCR3A and CXCR3B variants in benign and PTC tumors were determined by Western blot analysis using a monoclonal antibody (mAb) that discriminates CXCR3A and CXCR3B monomers by size. α-tubulin was used as a loading control. **(A)** Representative western blot. **(B)** Densitometric analysis showing the CXCR3A or CXCR3B/α-tubulin ratio obtained from B-CLT (n=28), benign tumors (B, n=28), nMPTC (n=19) and MPTC (n=24). **(C)** Relative fold change analysis showing the CXCR3A or CXCR3B/α-tubulin ratio normalized by an average ratio obtained from B-CLT (n=28) in benign tumors (B, n=28), nMPTC (n=19) and MPTC (n=24). **(D)** nMPTC tumors were categorized for the presence of thyroiditis. The CXCR3A or CXCR3B/α-tubulin ratio in 19 nMPTC tumors, with (n=6) or without (n=13) thyroiditis were compared to 28 B-CLT. **(E)** The CXCR3A or CXCR3B/α-tubulin ratio in B-CLT tissues without thyroiditis (B-CLT-T, n=21), thyroiditis samples (n=19) (see materials and methods), B (n=28) and nMPTC (n=18). **(F)** CXCR3A and CXCR3B levels were determined as in (B) and the CXCR3A/CXCR3B ratio (protein balance) was plotted. In all graphs, data is shown as mean ± s.e.m. Ratios were analyzed by Mann-Whitney test (^*^p<0.05, ^**^p<0.01, ^***^p<0.001).

Immunohistochemistry analysis revealed that total CXCR3 levels were increased in PTC (Figure 1), however, the antibody used in this analysis did not account changes in the CXCR3 variants profile. To compare the proteins levels of CXCR3A and CXCR3B in benign and malignant thyroid tissue, western blot analysis was performed by using a CXCR3 mAb (clone 1-C6) that recognizes CXCR3A (41 kDa) and CXCR3B (44 kDa) [75] as independent bands (Supplementary Figure 5). A representative western blot analysis is shown in Figure 3A. In B-CLT, benign and PTC, CXCR3A bands were frequently stronger than CXCR3B whereas in B-CLT CXCR3B expression was weakly detected. In benign tumors, nMPTC and MPTC tissues, both CXCR3 variants showed a significant increase when compared to B-CLT (CXCR3A: p=0.0050, p=0.0014 and p=0.0353; CXCR3B: p=0.001, p<0.0001 and p<0.0001) (Figure 3B). To investigate the profile of CXCR3 variants in tumor progression, CXCR3A and CXCR3B levels were compared between nMPTC and MPTC. Data showed that both variants were significantly downregulated in MPTC (CXCR3A: 1.1190±0.4086, p=0.0184; CXCR3B: 0.8669±0.3755, p=0.0205) (Figure 3B). In fact, the association of CXCR3A and CXCR3B protein levels with histopathological features of PTC, tested by UVA (univariate analysis), showed that CXCR3A and CXCR3B upregulation was associated with the absence of lymphnode metastasis in PTC (OR 0.19, 95% IC 0.05–0.78 for both) (Table 5).

**Table 5:** Association between histopathological features of PTC and CXCR3A or CXCR3B protein expression

	Tumor Size (≥ 2 cm)	Extrathyroid Extension	Lymphovascular Invasion	Lymphonodal Metastasis	Thyroiditis
**CXCR3A Expression**					
Odds Ratio	0.40	0.40	1.76	**0.19**	0.80
95% CI	0.07 - 2.19	0.09 - 1.76	0.39 - 7.99	**0.05 - 0.78**	0.20 - 3.27
**CXCR3B Expression**					
Odds Ratio	0.40	1.11	0.96	**0.19**	0.46
95% CI	0.07 - 2.19	0.29 - 4.31	0.20 - 4.57	**0.05 - 0.78**	0.10 - 2.07

Significative results are showed in bold font.

To analyze the relative fold changes of CXCR3 variants over B-CLT, CXCR3A and CXCR3B/α-tubulin ratio of each patient was divided by a B-CLT average ratio. Although in nMPTC, CXCR3B presented a higher fold change in protein levels (2.71) compared to CXCR3A (2.04), the latter remained as the predominant expressed isoform (Figure 3C). We also determined whether significant changes in CXCR3A and CXCR3B levels found in PTC could be related to the presence of thyroid-infiltrating lymphocytes. Interestingly, the increase in CXCR3A and CXCR3B protein levels in nMPTC was higher in patients without thyroiditis (CXCR3A: 1.7680±0.7505, p=0.0002; CXCR3B: 1.3480±0.5230, p<0.0001) than those with thyroiditis (CXCR3A: 1.2210±0.3714, p=0.0459; CXCR3B: 1.0460±0.5350 p=0.0024) (Figure 3D), suggesting that most of the increased protein levels of CXCR3A and CXCR3B might come from epithelial thyroid cells rather than from the inflammatory infiltrate in PTC.

It has been previously reported that the IFN-γ-inducible chemokines and their receptor CXCR3 potentiate the initiation of HT [13]. Our results showed an increased level of CXCR3 ligands in nMPTC tissues with thyroiditis, observation that strongly supports the notion that inflammation plays a key role in neoplastic transformation and cancer development through the CXCR3/ligand axis. To determine if changes in the CXCR3 expression profile are indeed associated to thyroid chronic inflammation, we analyzed the protein profile of CXCR3A and CXCR3B in tissues with thyroiditis and compared them to B-CLT, benign and nMPTC tumors. In this case, the contralateral tissue of benign tumors without thyroiditis (B-CLT-T) was used as the control tissue. Results showed a progressive increase in protein levels for CXCR3A and CXCR3B. Control tissue exhibited the lowest amount of protein followed by thyroiditis, benign and nMPTC tumors, the latter presenting the highest protein levels (Figure 3E). A significant increase of CXCR3B expression was found in thyroiditis compared to B-CLT (0.5747±0.433, p=0.0199), while both CXCR3 variants showed a statistical increment in nMPTC tumors compared to thyroiditis (CXCR3A: 1.593±0.7143, p=0.0171; CXCR3B: 1.240±0.5445 p=0.0004) (Figure 3E).

The analyses of protein balance (CXCR3A/CXCR3B ratio) in benign tumors and cancer thyroid tissues showed a predominant CXCR3A expression over the CXCR3B isoform (Figure 3F). A significant and progressive decrease in the protein ratio was observed from B-CLT to MPTC (Figure 3F) due to a greater fold change increase of the CXCR3B variant. A major decrease in the protein balance was observed when comparing B-CLT and benign nodules (p=0.0292), and between nMPTC (p=0.0005) or MPTC (p<0.0001) with B-CLT. No major difference in the protein ratio was found between nMPTC and MPTC. Thus, results suggest that changes in the ratio of the CXCR3 variants is associated to neoplastic transformation and tumor growth rather than tumor metastasis.

### CXCR3A is upregulated in papillary thyroid cancer epithelial cell line (TPC-1) and promotes proliferation in Nthy-ori-3-1 cells

**Figure 4:**
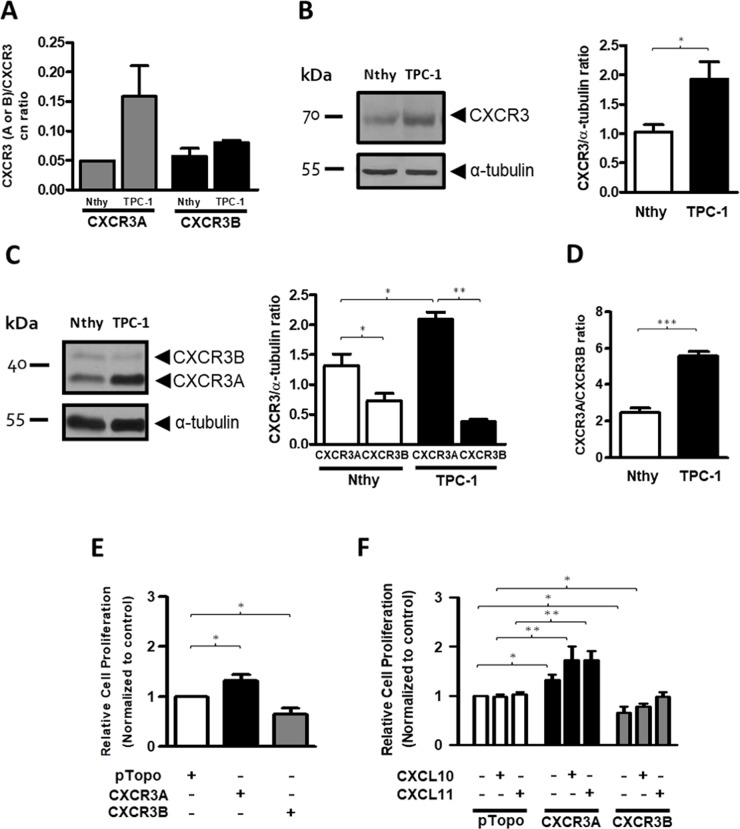
CXCR3A is upregulated in thyroid cancer cells (TPC-1) and promotes proliferation in the Nthy-ori-3-1cell line The figure shows the expression of CXCR3 variants in Nthy-ori-3-1 and TPC-1 cell lines. **(A)** CXCR3A and CXCR3B mRNA expression was determined by qPCR. Copy number (cn) ratio for CXCR3A and CXCR3B normalized by total CXCR3 receptor was analyzed in Nthy-ori-3-1 and TPC-1. **(B, C)** Western blot analyses of total CXCR3 receptor (B) and its variants (C) in Nthy-ori-3-1 and TPC-1 cells. Detection was achieved by using a CXCR3 polyclonal antibody (Abcam) and a monoclonal antibody (R&D Systems) respectively. Molecular weights are indicated in arrows and α-tubulin was used as loading control. *Left panel,* a representative experiment for each western blot analysis is displayed. *Right panel*, quantification of total CXCR3 receptor, CXCR3A and CXCR3B in Nthy-ori-3-1 and TPC-1 was performed by densitometric analysis. **(D)** CXCR3A/CXCR3B ratio in Nthy-ori-3-1 and TPC-1 cells. The results are shown as CXCR3, CXCR3A or CXCR3B/α-tubulin ratio. **(E)** Relative cell proliferation rate for cells transfected with empty vector (pTopo), CXCR3A or CXCR3B. **(F)** Relative cell proliferation rate for cells transfected with empty vector, CXCR3A or CXCR3B stimulated with CXCL10 or CXCL11. All experiments were performed in triplicate and in 3 independent experiments. In all graphs, data is shown as mean ± s.e.m and Mann-Whitney test was performed (^*^p<0.05, ^**^p<0.01, ^***^p<0.001).

Our results showed a major shift in CXCR3B expression than in CXCR3A. In order to clarify these seemingly contradictory findings and to better understand the CXCR3 variants profile in epithelial cells within PTC tissues, we analyzed their mRNA levels in Nthy-ori-3-1 and TPC-1 cell lines. CXCR3A copy number ratio in TPC-1 was increased by three-fold (0.158±0.089) compared to Nthy-ori-3-1 (0.048±0.0007), while CXCR3B levels were similar in both cell lines (0.0804±0.006 vs. 0.0569±0.0229) (Figure 4A). Compared to Nthy-ori-3-1, protein levels of total CXCR3 were significantly increased in TPC-1 (p=0.0162) (Figure 4B), but in both cell lines, CXCR3A was the most abundant variant (Figure 4C, left panel). Furthermore, higher CXCR3A levels were detected in TPC-1 (2.091±0.269, p=0.0303) when compared to Nthy-ori-3-1 (1.312±0.478) (Figure 4C, right panel), while the opposite was observed for CXCR3B levels which were subtly reduced in TPC-1 (0.374±0.096 vs. 0.724±0.359). Moreover, CXCR3B protein levels were significant lower than CXCR3A in Nthy-ori-3-1 and TPC-1 (p=0.0293 and p=0.0079 respectively) indicating that this cellular system recapitulates the CXCR3 profile progression found in control tissue and tumors (Figure 4C, right panel). Changes in the CXCR3A and CXCR3B profile in Nthy-ori-3-1 and TPC-1 cells, represented by CXCR3A/CXCR3B ratio (also defined as CXCR3A/CXCR3B balance) are shown in Figure 4D. CXCR3A/CXCR3B ratio in TPC-1 was increased by two folds (5.547±1.575) compared to Nthy-ori 3-1 (2.490±1.570) (Figure 4D). Since CXCR3A was upregulated in TPC-1 cells whereas CXCR3B expression was subtly diminished, but considering that CXCR3B showed higher fold change in protein levels than CXCR3A in nMPTC specimens, Nthy-ori 3-1 cells were transfected with empty vector, CXCR3A or CXCR3B. A significant increase in the relative cell proliferation rate was evidenced in CXCR3A-overexpressing cells (1.32±0.28, p=0.0313) when compared to the control (Figure 4E). In contrast, CXCR3B overexpression induced a significant decreased in cell growth (0.65±0.36, p=0.0391) (Figure 4E). When cells were stimulated with CXCL10 or CXCL11, a statistical and significant increase in the cell proliferation rate was observed in CXCR3A overexpressing cells (p=0.0036 and p=0.0014, respectively) while CXCR3B transfected cells stimulated with CXCL10 showed a significant decreased in cell division (p=0.0419) (Figure 4F).

### CXCL11 is the predominant secreted ligand in thyroid epithelia but only CXCL10 is significantly induced by IFN-ɣ in PTC cell line

**Figure 5:**
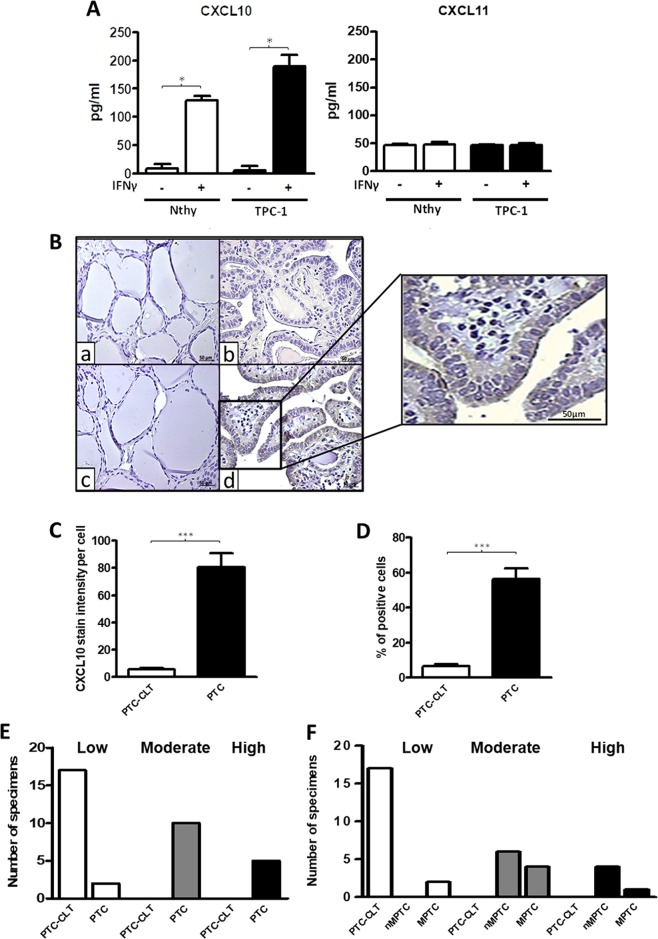
Increased CXCL10 levels in thyroid cell lines and in PTC tissues **(A)** Nthy-ori-3-1 and TPC-1 cells were incubated with or without IFN-ɣ for 24 h. in serum-free media and CXCL10 and CXCL11 levels in the supernatants were determined by ELISA. Secreted levels of CXCL10 and CXCL11 in basal and IFN-ɣ treated cells were quantified and plotted as bar graphs. Results are from three independent experiments performed in triplicate. Secretion levels are displayed as pg/mL for CXCL10 *(left panel)* and for CXCL11 *(right panel).* **(B)** Histological analysis of CXCL10 expression in PTC and their PTC-CLT. Representative photomicrographs from immunohistochemistry experiments for CXCL10 detection in 17 PTC and their contralateral tissues (PTC-CLT). PTC-CLT (a, c) and PTC (b, d) tissue samples incubated with CXCL10 antibody (c, d) or left without primary antibody (a, b) for background determination. Inset = Higher magnification of the area in the frame. Scale bar = 50 μm. Magnifications in 400x. **(C)** Quantitative analysis of CXCL10 staining intensity in 17 PTC tumors was performed as described in materials and methods. **(D)** Quantitative analysis of percentage of positive cells for CXCL10 immunostaining in 17 PTC tumors. **(E)** Frequency graph of the categories of the percentage of positive cells for CXCL10 immunostaining in 17 PTC tumors and 17 PTC-CLT. **(F)** Frequency graph of low, moderate or high percentage of positive cells for CXCL10 immunostaining in 10 non-metastatic PTC (nMPTC), 17 metastatic PTC (MPTC) and their contralateral tissue. Average staining intensity values and percentage of positive cells were normalized by corresponding total cell number in each slide and plotted as bar graphs. In all graphs, data is shown as mean ± s.e.m. Statistical analysis for chemokine secretion levels was performed using Mann-Whitney test and for CXCL10 immunohistochemistry was performed using a two-tailed paired t-test (^*^p<0.05, ^**^p<0.01, ^***^p<0.001).

Next we sought to evaluate whether IFN-ɣ could promote a differential response in secreted levels of CXCR3 ligands in Nthy-ori-3-1 and TPC-1 cells. Under serum free media conditions, we found that CXCL10 levels in Nthy-ori-3-1 and TPC-1 cells (8.276±19.94 and 5.642±15.64 pg/ml, respectively) were lower than that of CXCL11 (47.08±3.585 and 46.72±2.799 pg/ml respectively) (Figure 5A). No statistical differences were found for these ligands between both cells lines in starving media. In contrast, CXCL10 was robustly induced by IFN-ɣ and this increase was significantly higher in TPC-1 compared to Nthy-ori-3-1 (189.8±39.47 pg/ml, p=0.0286 vs. 129.6±12.41 pg/ml, p=0.0357). Therefore, results suggest that CXCL10 expression is highly induced by an inflammatory stimulus, such as IFN-ɣ, in normal and cancer thyroid epithelial cells.

### CXCL10 protein levels are upregulated in PTC

To determine whether CXCL10 expression partially originates from epithelial cells in PTC tumors, immunohistochemistry assays were performed. A major increase in CXCL10 immunoreactivity was detected in PTC epithelial cells compared to PTC-CLT (Figure 5B, section c and d). A higher magnification image of section d is shown in Figure 5B. Quantitative analysis showed a 16-fold increase in cell intensity per cell for CXCL10 expression in PTC (82.24±52.54 vs. 5.08±3.77, p<0.0001) (Figure 5C). No association was found between CXCL10 staining and other histopathological features (Table 6). The percentage of cells expressing CXCL10 as determined by immunostaining was higher in PTC (56.46%) than in PTC-CLT (6.74%, p<0.0001) (Figure 5D). The frequency graph of CXCL10 positive cells showed that most of PTC specimens presented moderate or high frequencies (% of CXCL10 immune reactive cells between 34-66% and 67-100%, respectively), whereas PTC-CLT cases were only distributed in low frequencies (% of CXCL10 immune reactive cells lower than 34%) (Figure 5E). When the CXCL10 frequency of positive cells was analyzed between MPTC and nMPTC specimens, cases with moderate frequency were very similar in both groups. In contrast, specimens with higher frequency of CXCL10 were more abundant in nMPTC than in MPTC (Figure 5F). CXCL10 expression was found in 100% of PTC specimens and all of them showed high intensity (data not shown).

**Table 6:** Association between histopathological features and CXCL10 immunoreactivity by univariate analysis

	Tumor Size (≥ 2 cm)	Extrathyroid Extension	Lymphovascular Invasion	Lymphonodal Metastasis	Thyroiditis
**Immunoreactivity**					
Odds Ratio	0.88	1.2	2.86	1.67	2.4
95% CI	0.11 - 7.11	0.16 - 8.80	0.24 - 33.90	0.21 - 13.22	0.30 - 19.04

Significative results are showed in bold font.

## DISCUSSION

It has been suggested that inflammation promotes an increased risk to neoplastic malignant transformation in thyroid tissues [18] and may be involved in thyroid cancer progression [20]. IFN-γ-mediated CXCL10 secretion is associated to thyroiditis and is closely related to thyroid tumors [49]. However, the role of CXCR3 spliced variants and their relation with thyroid cancer and inflammation has not been addressed. We report a progressive increase in protein balance of CXCR3 variants when comparing control tissue to benign tumors and malignant thyroid neoplasia where CXCR3A was always the predominant isoform. We also report that inflammation was conditioning for CXCR3 ligand increased availability in PTC. Finally, we show that CXCR3A and CXCL10 are highly expressed in non-metastatic PTC and in the PTC epithelial cell line TPC-1, consistent with a condition that may favor neoplastic transformation and tumor growth.

Our initial data showed increased protein levels of CXCR3 and CXCL10 in PTC. CXCR3 and CXCL10 immunoreactivity was mainly localized in epithelial cells within the tumor, but some minor staining was also observed in the inflammatory infiltrate. Thus, we cannot ignore a CXCR3A contribution from immune cells to the PTC-CXCR3A profile. In fact, a higher proportion of the CXCR3+/CCR5+ thyroid-infiltrating lymphocytes gained by fine needle aspiration biopsy has been recently reported in malignant nodules as compared to benign nodules [76] confirming some CXCR3 expression due to an inflammatory reaction surrounding thyroid carcinoma, as previously described [77]. However, our results suggest that the increase of CXCR3 expression in PTC is mainly due to an increased receptor expression in epithelial cells and not an exclusive contribution from the inflammatory infiltrate or stroma. Most of our PTC specimens with high CXCR3 staining were cases without thyroiditis and no major differences in CXCR3 immunoreactivity were found between PTC with or without thyroiditis. In addition, CXCR3A and CXCR3B protein levels in nMPTC were higher in patients without thyroiditis supporting that increased expression of CXCR3 is predominantly from epithelial cancer cells. In fact, we found increased protein levels for total receptor in TPC-1 when compared to Nthy-ori-3-1 cells. In ovarian cancer, the protumoral variant was observed in the inflammatory infiltrate, but was mainly detected in cancer cells [74]. Taken together, these results suggested that the increment of CXCR3 in PTC might be mainly epithelial with a minor contribution from Th-1 lymphocytes or stromal cells. Our findings prompted us to analyze the expression profile of CXCR3 in a papillary thyroid cancer cell line (TPC-1) as well as in benign and malignant thyroid neoplasms to determine if expression profiles are associated to thyroid malignant phenotypes.

Our results showed that in control tissue, if there is an imbalance of CXCR3 (favoring CXCR3A over CXCR3B levels), which gradually decreased as tissues becomes neoplastic (benign) and then malignant (nMPTC and MPTC). The increase of CXCR3A and CXCR3B protein levels between control and benign tumor tissue suggests a neoplastic transformation role for the CXCR3 variants profile. The balance of protein levels of CXCR3A and CXCR3B found in nMPTC suggest that similar levels for CXCR3 variant might have a role during malignant transformation or tumor growth. In benign tumors and PTC, proteins levels for CXCR3A and CXCR3B were significantly increased when compared to B-CLT, but CXCR3A was the most expressed variant with highest levels in nMPTC. Furthermore, since (i) CXCR3 staining was mainly observed in epithelial cells within the tumor, (ii) the protein levels for CXCR3A were significantly higher in TPC-1 than in Nthy-ori-3-1 and (iii) CXCR3B protein levels in TPC-1 were significantly lower than CXCR3A and (iv) CXCR3B levels in TPC-1 were lower than Nthy-ori-3-1), we propose that increased CXCR3 levels found in PTC by immunohistochemistry analysis correspond to the CXCR3A variant mainly expressed in PTC epithelial cells with a minor CXCR3A expression from inflammatory cells [74, 78, 79].

Despite that CXCR3A was predominant in PTC we detected an unexpected increase of CXCR3B. In TPC-1, CXCR3B protein levels were not increased when compared to Nthy-ori-3-1 cells. These results support that CXCR3B increment found in benign tumors or PTC may originate from other cell types within the tumor rather than epithelial cells. CXCR3B is primarily expressed in endothelial cells [46], in fibroblasts and T-lymphocytes but it has been found in several endocrine malignant neoplasia [80]. In epithelial breast cancer cells, decreased mRNA levels for CXCR3B were induced by upregulation of CXCL10 [67]. Consistently, we found increased CXCL10 at mRNA and protein levels in PTC tissues while CXCR3B mRNA levels were significantly downregulated. In fact, previous reports have shown that a significant proportion of freshly isolated PBMC (peripheral blood mononuclear cells) expressed CXCR3 at the plasma membrane while transcripts remained undetectable [81, 82]. Unexpectedly, CXCR3B proteins were increased in all tissues when compared to B-CLT. This may be due to a reduced receptor destination to proteasomes or lysosomes. Taken together, these results suggest a mechanism for CXCR3B mRNA downregulation induced by CXCL10 in PTC thyrocytes, reinforcing that CXCR3B protein increment found in PTC may not mainly come from tumoral epithelial cells. In ovarian and renal cancer, CXCR3B was preferentially detected in endothelial cells [46, 74], supporting the notion that CXCR3B activation may inhibit the PTC tumor associated angiogenesis. Other source for increased CXCR3B in PTC could be thyroid cancer stem cell-like populations, since CXCR3B has been found upregulated in this cells from breast cancer [70]. CXCR3B expression is frequently downregulated in several cancer types [67, 73, 74, 83], consistently with a reduced anti-proliferative and limited angiostatic activity [46]. Over-expression of CXCR3B in cancer cells significantly inhibited cell proliferation and promoted apoptosis [45, 74, 84] and in renal [57, 85] and gastric cancer [86] it was associated with tumor necrosis extension and immune-angiostatic activity respectively, demonstrating an anti-tumoral role for CXCR3B upregulation.

Changes in CXCR3 profile expression may be related to initial stages of cancer development or its progression to metastatic disease. CXCR3A has been associated to tumor progression in prostate, ovarian, breast and renal cancer [73, 74, 83]. When compared to nMPTC, a significant decrease of CXCR3A and CXCR3B was observed in MPTC but CXCR3A continued being the predominant variant. These results suggest that CXCR3A expression may not be associated to tumor thyroid dissemination. However, it has been reported that cells from metastatic primary tumors that have undergone epithelial–mesenchymal transition (EMT) display a reduced proliferation rate that is linked to dissemination and survival of metastatic cells [87] which would be consistent with a reduced CXCR3A expression. Further studies are necessary to understand how a weakened CXCR3A expression in MPTC could favor cell migration and tumor invasion.

Opposite effects for CXCR3 ligands depend on the array of spliced-variants and their localization (stroma and/or tumor cells) [80]. In nMPTC, CXCL10 and CXCL11 upregulation was associated to an increased CXCR3A expression, supporting a potential role for CXCR3A signaling during malignant neoplastic transformation. In fact, CXCR3A overexpressing cells promoted cell proliferation. Moreover, CXCL10 and CXCL11 induced significant increase in cell growth compared to cells transfected with empty vector. CXCL9, CXCL10 and CXCL11 has shown to induce cell proliferation on HMC, which selectively express CXCR3A [88, 89]. Likewise, CXCR3A overexpression in HEK293 cells promoted proliferation in basal and CXCL10 treated cells and PTX strongly reduced the proliferation in both conditions [46].

All CXCR3 chemokines were significantly reduced in MPTC when compared to nMPTC. The declining expression of CXCR3A and CXCR3B together with the low levels of CXCR3 ligands detected in MPTC, suggest that in cancer thyrocytes, CXCR3 signaling may not be involved in tumor dissemination and LNM.

Chronic inflammation in the tumor microenvironment plays a crucial role in tumorigenesis. Inflammatory cytokines and chemokines in tumor microenvironments attract immunosuppressive cells, and enhance EMT, tumor angiogenesis and cancer-stem cell formation. TNF-α is a master switch from chronic inflammation to cancer [90, 91]. Recent data has shown that TNF-α significantly increased the expression of CXCR2 and CXCR3 and their related ligands in renal cancer cells enhancing the migration, invasion and EMT [92]. Considering that in thyroid tissues, recruited Th-1 lymphocytes are responsible for enhanced IFN-γ and TNF-α production, which in turn stimulates CXCL10 secretion in thyroid cells perpetuating the autoimmune process [93], thus TNF-α stimulation of thyrocytes would clarify whether inflammatory microenvironment upregulates CXCR3 expression promoting thyroid tumorigenesis. Interestingly, CXCR3 ligands were statistically increased only in nMPTC patients with thyroiditis, indicating that the inflammation microenvironment is a prerequisite for increased CXCR3 ligand availability. In fact, CXCL10 was significantly induced by IFN-γ in Nthy-ori-3-1 and TPC-1, as has been previously reported in normal and PTC primary thyrocytes [53]. Inflammation promoted a switch on the CXCR3 ligand profile in normal and cancer epithelial cells, where CXCL10 became the predominant chemokine. Main cellular sources of CXCL10 in PTC tissues are Th-1 lymphocytes and mast cell infiltrate [94, 95], the later which has been associated with a pro-tumorigenic role in PTC [95, 96].

**Figure 6:**
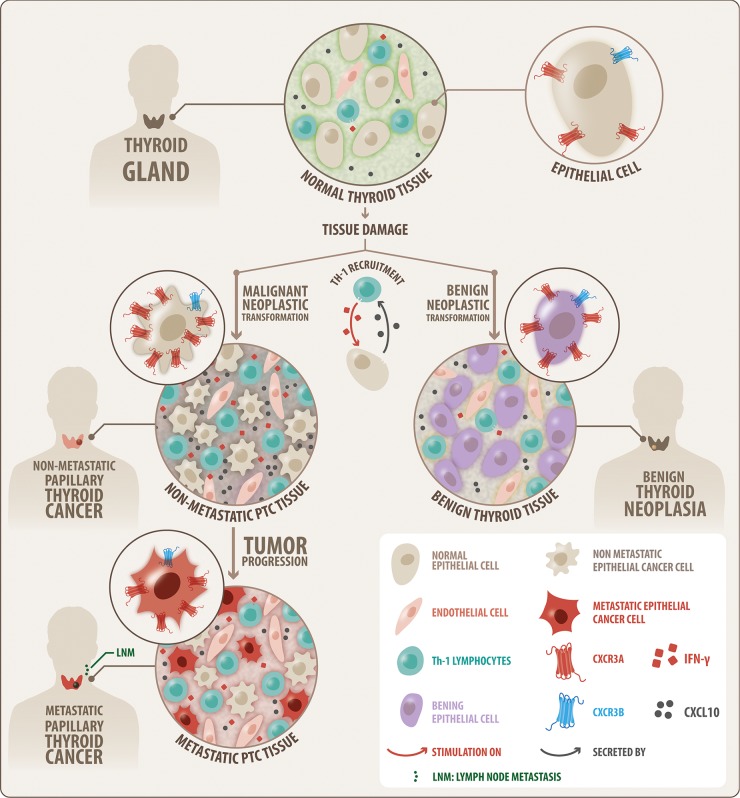
Schematic modeling of the CXCR3 variant expression profile that is associated to thyroid benign neoplasia and PTC A progressive increase in protein balance for CXCR3A (red) and CXCR3B (blue) from control tissue to benign and malignant (PTC) tumors is shown, with consistent predominance of CXCR3A. The increase of CXCR3A and CXCR3B in benign tumor suggests a neoplastic transformation role. Higher levels of CXCR3A in nMPTC suggest a role in malignant transformation or tumor growth. The decrease of CXCR3A and CXCR3B in MPTC compared to nMPTC suggests that CXCR3A may not be associated to thyroid tumor dissemination but with tumor growth. Inflammation was conditioning for CXCR3 ligand increased availability in PTC. CXCR3A and CXCL10 are highly expressed in nMPTC consistent with a condition that may favor neoplastic transformation and tumor growth.

The role of chemokines and their receptors in malignant transformation in the tumor microenvironment is still debatable. Chronic inflammation causes DNA damage in proliferating cells [32, 97, 98] leading to neoplastic transformation [32]. CXCL10 is member of the interferon-related DNA damage signature, linking CXCL10 expression with pro-tumorigenic functions. Inflammation may promote CXCR3 upregulation in PTC by inducing its promoter demethylation [99] or by demethylation of intron CpG sites resulting in increased CXCR3A levels in cancer cells [61, 100]. In damaged thyroid tissue with cytokines surrounding the premalignant lesion, early recruitment and activation of Th-1 leukocytes and mast cells, may lead to constitutive secretion of CXCL10 that in turn promotes, by paracrine or autocrine manner, thyrocytes (and other cells) survival through CXCR3A signaling. We propose that persistent inflammation and sustained CXCL10 secretion, may upregulate CXCR3A in epithelial cells (Figure 6) promoting the malignant neoplastic transformation through constitutive CXCR3A-CXCL10 signaling. Then, in malignant tissue, tumor growth and cell survival may be supported by CXCR3A-CXCL10 axis, contributing to thyroid cancer progression but not to tumor dissemination. The function of increased CXCR3B expression could be related to a decreased tumor associated angiogenesis and to tumor dependent immunity. Further studies are pending to understand the mechanism underlying the increased CXCR3A and CXCR3B expression within PTC tissues and their role in cancer development and progression.

In conclusion, to our knowledge, this is the first report showing a switch in the CXCR3A/CXCR3B balance in PTC and its association with inflammation and thyroid cancer progression. The current study shows that CXCR3A and CXCL10 are highly expressed in PTC and in a PTC derived epithelial cell line (TPC-1), consistent with a condition that may favor tumor development and progression. In addition, CXCL10 is strongly induced by IFN-ɣ in CXCR3A over-expressing cells (TPC-1) suggesting that CXCR3A-CXCL10 enhanced signaling might induce tumor progression promoting signals in PTC.

## MATERIALS AND METHODS

### Patients and tissue samples

Surgical tissue specimens included malignant (PTC, with or without LNM (metastatic PTC, (MPTC) and non-metastatic (nMPTC) respectively)) and benign (B) thyroid tumors. Fresh benign and PTC tumors and their respective contralateral tissues (B-CLT and PTC-CLT) were obtained from patients undergoing total thyroidectomy. Patients signed an informed consent approved by the ethics committee of the Pontificia Universidad Católica de Chile according to the principles expressed in the declaration of Helsinki. Tissue samples were divided and placed in 4% paraformaldehyde or protein lysis solution, or preserved in a RNA stabilization solution (Ambion, Austin TX). Inclusion criteria were patients at least 18 years old and with a confirmed benign or malignant diagnosis by histopathological examination (biopsy report). Exclusion criteria were patients with follicular, medullary and anaplastic thyroid cancer. Thyroid biopsy chart showing benign and PTC cases distribution for IHQ, qPCR and WB analysis is shown in Supplementary Figure 3. Tissue characterization is described as follows; B-CLT: contralateral tissue of benign tumors considered as non-neoplastic and non-malignant tissue; PTC-CLT: contralateral tissue of PTC tumors; nMPTC-CLT: contralateral tissue of non-metastatic PTC; MPTC-CLT: contralateral tissue from metastatic PTC. All contralateral tissues from PTC tumors were considered as non-neoplastic and non-malignant tissue; B: benign thyroid tumor considered as neoplastic and non-malignant tissue; PTC: cancer tissue considered as neoplastic and malignant tissue.

PTC tumors included PTC - UV (Usual variant), PTC - FV (Follicular variant), PTC - HCV (Hurthle Cell variant), PTC - TCV (Tall Cell variant), and PTC-DSV (Diffuse-Sclerosant variant). Benign thyroid tumors included HP (Hyperplastic nodules) and FA (Follicular adenomas). As thyroiditis samples, the contralateral thyroid tissue of 7 cases of benign tumors and the contralateral thyroid tissues of 11 cases of PTC tumors, which presented severe or moderate thyroiditis in the biopsy report were considered (Supplementary Figure 3). To establish our gold standard control tissue, we compared the mRNA levels of CXCR3A and CXCR3B between B-CLT and PTC-CLT tissues. Further, sub analysis comparing the expression for CXCR3 variants was performed between nMPTC-CLT and MPTC-CLT. Expression of CXCR3A was significantly increased in nMPTC-CLT when compared to B-CLT, whereas CXCR3B levels were only slightly increased (Supplementary Figure 4), indicating that PTC-CLT may not represent a qualified control. Therefore, we considered B-CLT as the gold standard for non-malignant tissue (control) in the mRNA and protein profiles analyses.

### Cell cultures

Nthy-ori-3-1, a normal human thyroid follicular epithelial immortalized cell line [101] was purchased from ECACC (Wiltshire, UK). TPC-1, a human PTC cell line derived from a PTC that expresses the RET/PTC-1 oncogene [102], was kindly provided by Dr. J. A. Fagin (Memorial Sloan Kettering Cancer Center, New York, NY). Cells were grown in a humidified atmosphere containing 5% CO_2_ at 37°C in RPMI 1640, 10% fetal bovine serum, penicillin-streptomycin (100 U/ml) (Invitrogen-GIBCO, MD, USA). Nthy-ori-3-1 culture media was supplemented with L-glutamine 2 mM. Cell lines were authenticated and routinely tested for bacterial and Mycoplasma contamination.

### Real-time PCR (qPCR) reaction and qPCR analyses

Expression of CXCR3 variants and ligands was analyzed by real time RT-PCR (qPCR) using Brilliant II SYBR® Green QPCR Master Mix (Stratagene) following the manufacturer instructions. All reactions were performed in triplicate using 10 ηg of cDNA. ACTB and RNA18S were used as control genes. Primer sequences for CXCR3 variants and their ligands were designed using the NCBI's Primer BLAST (http://www.ncbi.nlm.nih.gov/tools/primer-blast) and are listed in Table 4. Ct values were analyzed by Pfaffl's relative quantification model [103] presented as relative fold changes of mRNA amount in target tissue respect to reference tissue (Supplementary Materials).

### Immunohistochemistry

CXCR3 and CXCL10 protein levels in tissue samples were analyzed in formalin-fixed, paraffin-embedded samples obtained from PTC tumors and their contralateral tissue (PTC-CLT). Clinical information of PTC specimens analyzed by immunohistochemistry is summarized in Table 1. Primary mouse monoclonal (mAb) anti-human CXCR3 antibody (antibody that not account to CXCR3 variant profile due an almost complete overlap between variants aminoacid sequences) [104] (Abcam, ab64714) and polyclonal (pAb) anti-human CXCL10 antibody [67] (R&D Systems, AF-266-NA) were used. Further details of the procedure are described in Supplementary Materials.

The immunoreactivity intensity values were determined using the Image-Pro Plus program version 6.0 (Media Cybernetic Inc.) and the staining intensity of CXCR3 and CXCL10 in tumor tissue was normalized by total number of nuclei counted in each microphotograph and scored. To quantify immunoreactivity, 3 representative digital microphotographs were taken from sections of tumor and matched non-neoplastic tissue. The average normalized values were plotted and referred as intensity of CXCR3 or CXCL10 per cell. Frequency graphs for CXCR3 and CXCL10 were obtained by counting the total immunoreactive positive cells in at least 3 fields of each slide. Percentage of positive cells was obtained by dividing the number of stained cells by total cell number in each field. The percentage of positive cells of CXCR3 or CXCL10 in tumor tissue or in the PTC-CLT was scored based on the following criteria: low: (0-33% of positive cells), moderate (34-66% of positive cells) and high (67-100%). To assess the best cutoff value for the Odds Ratio analysis, a receiver operating characteristic (ROC) curve was constructed and analyzed (data not shown). Optimal sensitivity to specificity ratios were observed using the cutoff values of 90% of positive cells for CXCR3 and 58% of positive cells for CXCL10 and the immunoreactivity values of 493 for CXCR3 and 58.8 for CXCL10.

### Western blot

Western blot assays were performed for CXCR3 and their splicing variants expression analysis in benign, PTC tissues and thyroid cell lines. Primary mouse mAb for total CXCR3 [104] (Abcam, ab64714), mouse pAb for CXCR3 variants [74] (R&D Systems, MAB160), and mouse mAb for α-tubulin [105] (SIGMA, T9026) were used (Supplementary Materials).

### Quantitative detection of chemokines

Cells plated in 6 cm dishes were grown to 70% confluence. After 24 hours, cells were washed, cultured in serum-deprived media and treated with IFN-ɣ (10000 U/mL) for 24 hours. Control cells were maintained in serum-deprived media. Supernatants were harvested and cleared by centrifugation at 800 *g* at 4°C. Chemokine concentration was determined in triplicates by quantitative immunoassay ELISA kit (QuantiKine ELISA kit; R&D Systems) following the manufacturer's instructions.

### Plasmids

Plasmids containing the complete open reading frame of CXCR3A or CXCR3B genes were obtained by isolating the human sequences from benign patients by RT-PCR reaction. PCR fragments were then cloned into pcDNA 3.1/V5-His © TOPO ® TA (ptopo, Invitrogene)(USA). Variants sequences were under the control of CMV and T7 polymerase and alternatively fused to the V5 epitope, adding 45 extra aminoacids. pmCherry-V5 plasmid was a gift from Dr. R. Fuentealba (Universidad Autonoma, Chile).

### Transfection

Nthy-ori 3-1 cells (2×10^6^ cells/plate) were transfected with 15 μg of plasmid DNA into 100 mm plates with lipofectamine 3000 reagent (Invitrogen Inc., USA), and cultured at 37°C in an atmosphere of 5% CO_2_ for 48 h. Hela cells (1.5×10^5^ cells/plate) were transfected with 1 μg of plasmid DNA into 30 mm plates with Fugene 6 reagent (Promega, USA), and cultured at 37°C in an atmosphere of 5% CO_2_ for 24 h.

### MTT assays

Cell proliferation was measured by MTT cell proliferation assay (Trevigen, Gaithersburg, USA). Following transfection cells were plated (3×10^3^ cells/well) in 96-well plates with no phenol red RPMI medium mixed with 10% fetal bovine serum and then cultured at 37°C for 24 h. Then CXCL10 and CXCL11 were added at 100 ng/mL and the cells were cultured for 48 h and 10 μl of MTT reagent was added to each well. When purple crystals of formazan became visible under the microscope, 100 μl of detergent reagent was added, and the cells were incubated for 2 h. Absorbance of cells in each well was observed at 570 nm under an absorption spectrophotometer (Autobio Labtec, China) and corrected against blanks (culture medium). Cells transfected with empty vector (pTopo) were considered as control. All experiments were conducted independently for 3 times. The reading at 570 nm is directly proportional to cell proliferation (number of viable cells).

### *In vitro* transcription

The vectors were digested with PmeI (#ER1341, ThermoFisher Scientific) and the RNAs were synthesized in a 50 μl *in vitro* transcription reaction for 2 h at 37°C using T7 RNA polymerase (#EP0111, Thermo Scientific, ThermoFisher Scientific), 5 mM rNTPs, 1X Ribomax transcription buffer (80 mM Hepes-KOH pH 7.5, 24 mM MgCl_2_, 2 mM spermidine, 40 mM DTT) and 20 U of RNAsin (#E00382, Thermo Scientific, ThermoFisher Scientific). Upon synthesis, RNA was treated with 1 U of RQ1 DNase (#M610A, Promega) for 30 min at 37°C. RNA was precipitated for 2 h at -20°C with 2.5 M LiCl, centrifuged at 16,000 g for 30 min at 4°C, washed with 70% ethanol and resuspended in 25 μl of nuclease-free water. RNA concentrations were determined spectrophotometrically (NanoDrop Technology, Wilmington, DE), and RNA integrity was monitored by electrophoresis on agarose gels.

### *In vitro* translation

*In vitro* translation reactions were carried out in nuclease-treated rabbit reticulocyte lysate (RRL; #L4960, Promega, Madison, WI) according to the manufacturer instructions using 1 μg of RNA in each reaction at 70% v/v of RRL supplemented with 0.1 mM of an amino acid mixture minus leucine (#L9951, Promega), 0.1 mM of an amino acid mixture minus methionine (#L9961, Promega) and 40 U of RNAsin (#E00382, Thermo Scientific, ThermoFisher Scientific). The final volume in all reactions was 50 μL. As a negative control, a reaction without RNA was used. All the reactions were carried out during 90 minutes at 30°C and after that, the reactions were stopped placing the tubes at -20°C.

### Statistical analysis

Statistical significance for experiments in which data were compared to a standard or predetermined value (Pfaffl's graphs), was performed by using a Wilcoxon test (for non-parametrical data) or a two-tailed t-test (for parametrical data). In experiments where data between several groups were compared, Mann-Whitney test (for non-parametrical data) or a paired or unpaired two-tailed t-test (for parametrical data) was performed. Data are presented as means ± standard error of the mean (s.e.m.) with sample size (n) indicating the number of samples (patients) or (n) indicating the number of independent experiments (cell lines). Significance of mean comparison was considered when p values were less than 0.05. All data were tested for normality with D’Agostino and Pearson omnibus test. The association between the protein expression of CXCR3, CXCR3A, CXCR3B and CXCL10 with each histopathological feature of PTC was determined by the calculation of the Odds Ratio (OR) by univariate analysis. The 95% of the confidence interval was also calculated for OR, resulting statistically important when did not cross the unit value. All analyses were performed using GraphPad Prism 5, version 5.01 ® software (GraphPad Prism, Inc). No statistical method was used to predetermine sample size therefore it was estimated according to biopsy availability.

## SUPPLEMENTARY MATERIALS FIGURES



## Abbreviations

B: Benign
PTC: papillary thyroid cancer
CLT: contralateral tissue
B-CLT: benign contralateral tissue
PTC-CLT: PTC contralateral tissue
nMPTC: nonmetastatic PTC
nMPTC-CLT: nMPTC contralateral tissue
MPTC: metastatic PTC
MPTC-CLT: MPTC contralateral tissue
CXCR3: chemokine receptor 3 with C-X-C motive
CXCL4: Chemokine 4 with C-X-C motive
CXCL9: Chemokine 9 with C-X-C motive
CXCL10: Chemokine 10 C-X-C motive
CXCL11: Chemokine 11 with C-X-C motive
CXCR3A: CXCR3 splice variant A
CXCR3B: CXCR3 splice variant B
IFN-γ: interferon gamma
TNFα: tumor necrosis factor alpha
Th-1: helper T lymphocyte type 1

## References

[1] Cramer JD, Fu P, Harth KC, Margevicius S, Wilhelm SM (2010). Analysis of the rising incidence of thyroid cancer using the surveillance, epidemiology and end results national cancer data registry. Surgery.

[2] Davies L, Welch HG (2006). Increasing incidence of thyroid cancer in the united states, 1973-2002. JAMA.

[3] Pellegriti G, Frasca F, Regalbuto C, Squatrito S, Vigneri R (2013). Worldwide increasing incidence of thyroid cancer: update on epidemiology and risk factors. J Cancer Epidemiol.

[4] La Vecchia C, Malvezzi M, Bosetti C, Garavello W, Bertuccio P, Levi F, Negri E (2015). Thyroid cancer mortality and incidence: a global overview. Int J Cancer.

[5] Rahib L, Smith BD, Aizenberg R, Rosenzweig AB, Fleshman JM, Matrisian LM (2014). Projecting cancer incidence and deaths to 2030: the unexpected burden of thyroid, liver, and pancreas cancers in the united states. Cancer Res.

[6] Cooper DS, Doherty GM, Haugen BR, Kloos RT, Lee SL, Mandel SJ, Mazzaferri EL, McIver B, Pacini F, Schlumberger M, Sherman SI, Steward DL, Tuttle RM (2009). Revised american thyroid association management guidelines for patients with thyroid nodules and differentiated thyroid cancer. Thyroid.

[7] Tuttle RM, Tala H, Shah J, Leboeuf R, Ghossein R, Gonen M, Brokhin M, Omry G, Fagin JA, Shaha A (2010). Estimating risk of recurrence in differentiated thyroid cancer after total thyroidectomy and radioactive iodine remnant ablation: using response to therapy variables to modify the initial risk estimates predicted by the new American Thyroid Association staging system. Thyroid.

[8] Liotti F, Visciano C, Melillo RM (2012). Inflammation in thyroid oncogenesis. Am J Cancer Res.

[9] Colotta F, Allavena P, Sica A, Garlanda C, Mantovani A (2009). Cancer-related inflammation, the seventh hallmark of cancer: links to genetic instability. Carcinogenesis.

[10] Loh KC, Greenspan FS, Dong F, Miller TR, Yeo PP (1999). Influence of lymphocytic thyroiditis on the prognostic outcome of patients with papillary thyroid carcinoma. J Clin Endocrinol Metab.

[11] Ugolini C, Basolo F, Proietti A, Vitti P, Elisei R, Miccoli P, Toniolo A (2007). Lymphocyte and immature dendritic cell infiltrates in differentiated, poorly differentiated, and undifferentiated thyroid carcinoma. Thyroid.

[12] Larson SD, Jackson LN, Riall TS, Uchida T, Thomas RP, Qiu S, Evers BM (2007). Increased incidence of well-differentiated thyroid cancer associated with hashimoto thyroiditis and the role of the PI3k/Akt pathway. J Am Coll Surg.

[13] Repplinger D, Bargren A, Zhang YW, Adler JT, Haymart M, Chen H (2008). Is hashimoto's thyroiditis a risk factor for papillary thyroid cancer?. J Surg Res.

[14] Liu CL, Cheng SP, Lin HW, Lai YL (2014). Risk of thyroid cancer in patients with thyroiditis: a population-based cohort study. Ann Surg Oncol.

[15] Walker RP, Paloyan E (1990). The relationship between hashimoto's thyroiditis, thyroid neoplasia, and primary hyperparathyroidism. Otolaryngol Clin North Am.

[16] Lun Y, Wu X, Xia Q, Han Y, Zhang X, Liu Z, Wang F, Duan Z, Xin S, Zhang J (2013). Hashimoto's thyroiditis as a risk factor of papillary thyroid cancer may improve cancer prognosis. Otolaryngol Head Neck Surg.

[17] Ott RA, Calandra DB, McCall A, Shah KH, Lawrence AM, Paloyan E (1985). The incidence of thyroid carcinoma in patients with hashimoto's thyroiditis and solitary cold nodules. Surgery.

[18] Guarino V, Castellone MD, Avilla E, Melillo RM (2010). Thyroid cancer and inflammation. Mol Cell Endocrinol.

[19] Cunha LL, Marcello MA, Ward LS (2014). The role of the inflammatory microenvironment in thyroid carcinogenesis. Endocr Relat Cancer.

[20] Ward LS (2014). Immune response in thyroid cancer: widening the boundaries. Scientifica (Cairo).

[21] Okayasu I (1997). The relationship of lymphocytic thyroiditis to the development of thyroid carcinoma. Endocr Pathol.

[22] Antonaci A, Consorti F, Mardente S, Giovannone G (2009). Clinical and biological relationship between chronic lymphocytic thyroiditis and papillary thyroid carcinoma. Oncol Res.

[23] Singh B, Shaha AR, Trivedi H, Carew JF, Poluri A, Shah JP (1999). Coexistent hashimoto's thyroiditis with papillary thyroid carcinoma: impact on presentation, management, and outcome. Surgery.

[24] Kebebew E, Treseler PA, Ituarte PH, Clark OH (2001). Coexisting chronic lymphocytic thyroiditis and papillary thyroid cancer revisited. World J Surg.

[25] Borrello MG, Alberti L, Fischer A, Degl’innocenti D, Ferrario C, Gariboldi M, Marchesi F, Allavena P, Greco A, Collini P, Pilotti S, Cassinelli G, Bressan P (2005). Induction of a proinflammatory program in normal human thyrocytes by the RET/PTC1 oncogene. Proc Natl Acad Sci U S A.

[26] Castellone MD, Guarino V, De Falco V, Carlomagno F, Basolo F, Faviana P, Kruhoffer M, Orntoft T, Russell JP, Rothstein JL, Fusco A, Santoro M, Melillo RM (2004). Functional expression of the CXCR4 chemokine receptor is induced by RET/PTC oncogenes and is a common event in human papillary thyroid carcinomas. Oncogene.

[27] Melillo RM, Castellone MD, Guarino V, De Falco V, Cirafici AM, Salvatore G, Caiazzo F, Basolo F, Giannini R, Kruhoffer M, Orntoft T, Fusco A, Santoro M (2005). The RET/PTC-RAS-BRAF linear signaling cascade mediates the motile and mitogenic phenotype of thyroid cancer cells. J Clin Invest.

[28] Puxeddu E, Knauf JA, Sartor MA, Mitsutake N, Smith EP, Medvedovic M, Tomlinson CR, Moretti S, Fagin JA (2005). RET/PTC-induced gene expression in thyroid PCCL3 cells reveals early activation of genes involved in regulation of the immune response. Endocr Relat Cancer.

[29] Russell JP, Shinohara S, Melillo RM, Castellone MD, Santoro M, Rothstein JL (2003). Tyrosine kinase oncoprotein, RET/PTC3, induces the secretion of myeloid growth and chemotactic factors. Oncogene.

[30] De Falco V, Guarino V, Avilla E, Castellone MD, Salerno P, Salvatore G, Faviana P, Basolo F, Santoro M, Melillo RM (2007). Biological role and potential therapeutic targeting of the chemokine receptor CXCR4 in undifferentiated thyroid cancer. Cancer Res.

[31] Muzza M, Degl’Innocenti D, Colombo C, Perrino M, Ravasi E, Rossi S, Cirello V, Beck-Peccoz P, Borrello MG, Fugazzola L (2010). The tight relationship between papillary thyroid cancer, autoimmunity and inflammation: clinical and molecular studies. Clin Endocrinol (Oxf).

[32] Lu H, Ouyang W, Huang C (2006). Inflammation, a key event in cancer development. Mol Cancer Res.

[33] Mantovani A, Allavena P, Sica A, Balkwill F (2008). Cancer-related inflammation. Nature.

[34] Lazebnik Y (2010). What are the hallmarks of cancer?. Nat Rev Cancer.

[35] Dhawan P, Richmond A (2002). Role of cxcl1 in tumorigenesis of melanoma. J Leukoc Biol.

[36] Skinnider BF, Kapp U, Mak TW (2002). The role of interleukin 13 in classical hodgkin lymphoma. Leuk Lymphoma.

[37] Balkwill FR (2012). The chemokine system and cancer. J Pathol.

[38] Kato M, Kitayama J, Kazama S, Nagawa H (2003). Expression pattern of CXC chemokine receptor-4 is correlated with lymph node metastasis in human invasive ductal carcinoma. Breast Cancer Res.

[39] Raman D, Baugher PJ, Thu YM, Richmond A (2007). Role of chemokines in tumor growth. Cancer Lett.

[40] Strieter RM, Burdick MD, Gomperts BN, Belperio JA, Keane MP (2005). CXC chemokines in angiogenesis. Cytokine Growth Factor Rev.

[41] Tanaka K, Kurebayashi J, Sohda M, Nomura T, Prabhakar U, Yan L, Sonoo H (2009). The expression of monocyte chemotactic protein-1 in papillary thyroid carcinoma is correlated with lymph node metastasis and tumor recurrence. Thyroid.

[42] Balkwill F, Mantovani A (2001). Inflammation and cancer: back to virchow?. Lancet.

[43] Vicari AP, Caux C (2002). Chemokines in cancer. Cytokine Growth Factor Rev.

[44] Wu ST, Sun GH, Hsu CY, Huang CS, Wu YH, Wang HH, Sun KH (2011). Tumor necrosis factor-α induces epithelial-mesenchymal transition of renal cell carcinoma cells via a nuclear factor kappa B-independent mechanism. Exp Biol Med (Maywood).

[45] Kouroumalis A, Nibbs RJ, Aptel H, Wright KL, Kolios G, Ward SG (2005). The chemokines CXCL9, CXCL10, and CXCL11 differentially stimulate g alpha i-independent signaling and actin responses in human intestinal myofibroblasts. J Immunol.

[46] Lasagni L, Francalanci M, Annunziato F, Lazzeri E, Giannini S, Cosmi L, Sagrinati C, Mazzinghi B, Orlando C, Maggi E, Marra F, Romagnani S, Serio M (2003). An alternatively spliced variant of CXCR3 mediates the inhibition of endothelial cell growth induced by IP-10, Mig, and I-TAC, and acts as functional receptor for platelet factor 4. J Exp Med.

[47] Garcia-Lopez MA, Sanchez-Madrid F, Rodriguez-Frade JM, Mellado M, Acevedo A, Garcia MI, Albar JP, Martinez C, Marazuela M (2001). CXCR3 chemokine receptor distribution in normal and inflamed tissues: expression on activated lymphocytes, endothelial cells, and dendritic cells. Lab Invest.

[48] Ruffilli I, Ferrari SM, Colaci M, Ferri C, Fallahi P, Antonelli A (2014). IP-10 in autoimmune thyroiditis. Horm Metab Res.

[49] Rotondi M, Coperchini F, Pignatti P, Sideri R, Groppelli G, Leporati P, La Manna L, Magri F, Mariotti S, Chiovato L (2013). Interferon-gamma and tumor necrosis factor-alpha sustain secretion of specific CXC chemokines in human thyrocytes: a first step toward a differentiation between autoimmune and tumor-related inflammation?. J Clin Endocrinol Metab.

[50] Rotondi M, Chiovato L, Romagnani S, Serio M, Romagnani P (2007). Role of chemokines in endocrine autoimmune diseases. Endocr Rev.

[51] Antonelli A, Ferrari SM, Fallahi P, Ghiri E, Crescioli C, Romagnani P, Vitti P, Serio M, Ferrannini E (2010). Interferon-alpha, -beta and -gamma induce CXCL9 and CXCL10 secretion by human thyrocytes: modulation by peroxisome proliferator-activated receptor-gamma agonists. Cytokine.

[52] Antonelli A, Ferrari SM, Mancusi C, Mazzi V, Pupilli C, Centanni M, Ferri C, Ferrannini E, Fallahi P (2013). Interferon-alpha, -beta and -gamma induce CXCL11 secretion in human thyrocytes: modulation by peroxisome proliferator-activated receptor gamma agonists. Immunobiology.

[53] Antonelli A, Ferrari SM, Fallahi P, Frascerra S, Piaggi S, Gelmini S, Lupi C, Minuto M, Berti P, Benvenga S, Basolo F, Orlando C, Miccoli P (2009). Dysregulation of secretion of CXC alpha-chemokine CXCL10 in papillary thyroid cancer: modulation by peroxisome proliferator-activated receptor-gamma agonists. Endocr Relat Cancer.

[54] Arenberg DA, Kunkel SL, Polverini PJ, Morris SB, Burdick MD, Glass MC, Taub DT, Iannettoni MD, Whyte RI, Strieter RM (1996). Interferon-gamma-inducible protein 10 (IP-10) is an angiostatic factor that inhibits human non-small cell lung cancer (NSCLC) tumorigenesis and spontaneous metastases. J Exp Med.

[55] Goldberg-Bittman L, Neumark E, Sagi-Assif O, Azenshtein E, Meshel T, Witz IP, Ben-Baruch A (2004). The expression of the chemokine receptor CXCR3 and its ligand, CXCL10, in human breast adenocarcinoma cell lines. Immunol Lett.

[56] Goldberg-Bittman L, Sagi-Assif O, Meshel T, Nevo I, Levy-Nissenbaum O, Yron I, Witz IP, Ben-Baruch A (2005). Cellular characteristics of neuroblastoma cells: regulation by the ELR --CXC chemokine CXCL10 and expression of a CXCR3-like receptor. Cytokine.

[57] Suyama T, Furuya M, Nishiyama M, Kasuya Y, Kimura S, Ichikawa T, Ueda T, Nikaido T, Ito H, Ishikura H (2005). Up-regulation of the interferon gamma (IFN-gamma)-inducible chemokines IFN-inducible T-cell alpha chemoattractant and monokine induced by IFN-gamma and of their receptor CXC receptor 3 in human renal cell carcinoma. Cancer.

[58] Belperio JA, Keane MP, Arenberg DA, Addison CL, Ehlert JE, Burdick MD, Strieter RM (2000). CXC chemokines in angiogenesis. J Leukoc Biol.

[59] Strieter RM, Belperio JA, Phillips RJ, Keane MP (2004). CXC chemokines in angiogenesis of cancer. Semin Cancer Biol.

[60] Salcedo R, Oppenheim JJ (2003). Role of chemokines in angiogenesis: CXCL12/SDF-1 and CXCR4 interaction, a key regulator of endothelial cell responses. Microcirculation.

[61] Ma B, Khazali A, Wells A (2015). CXCR3 in carcinoma progression. Histol Histopathol.

[62] Kawada K, Sonoshita M, Sakashita H, Takabayashi A, Yamaoka Y, Manabe T, Inaba K, Minato N, Oshima M, Taketo MM (2004). Pivotal role of CXCR3 in melanoma cell metastasis to lymph nodes. Cancer Res.

[63] Robledo MM, Bartolome RA, Longo N, Rodriguez-Frade JM, Mellado M, Longo I, Van Muijen GN, Sanchez-Mateos P, Teixido J (2001). Expression of functional chemokine receptors CXCR3 and CXCR4 on human melanoma cells. J Biol Chem.

[64] Soejima K, Rollins BJ (2001). A functional IFN-gamma-inducible protein-10/CXCL10-specific receptor expressed by epithelial and endothelial cells that is neither CXCR3 nor glycosaminoglycan. J Immunol.

[65] Billiau A, Matthys P (2001). Modes of action of freund's adjuvants in experimental models of autoimmune diseases. J Leukoc Biol.

[66] Janatpour MJ, Hudak S, Sathe M, Sedgwick JD, McEvoy LM (2001). Tumor necrosis factor-dependent segmental control of MIG expression by high endothelial venules in inflamed lymph nodes regulates monocyte recruitment. J Exp Med.

[67] Datta D, Flaxenburg JA, Laxmanan S, Geehan C, Grimm M, Waaga-Gasser AM, Briscoe DM, Pal S (2006). Ras-induced modulation of CXCL10 and its receptor splice variant CXCR3-B in MDA-MB-435 and MCF-7 cells: relevance for the development of human breast cancer. Cancer Res.

[68] Datta D, Banerjee P, Gasser M, Waaga-Gasser AM, Pal S (2010). CXCR3-B can mediate growth-inhibitory signals in human renal cancer cells by down-regulating the expression of heme oxygenase-1. J Biol Chem.

[69] Giuliani N, Bonomini S, Romagnani P, Lazzaretti M, Morandi F, Colla S, Tagliaferri S, Lasagni L, Annunziato F, Crugnola M, Rizzoli V (2006). CXCR3 and its binding chemokines in myeloma cells: expression of isoforms and potential relationships with myeloma cell proliferation and survival. Haematologica.

[70] Li Y, Reader JC, Ma X, Kundu N, Kochel T, Fulton AM (2015). Divergent roles of CXCR3 isoforms in promoting cancer stem-like cell survival and metastasis. Breast Cancer Res Treat.

[71] Utsumi T, Suyama T, Imamura Y, Fuse M, Sakamoto S, Nihei N, Ueda T, Suzuki H, Seki N, Ichikawa T (2014). The association of CXCR3 and renal cell carcinoma metastasis. J Urol.

[72] Wightman SC, Uppal A, Pitroda SP, Ganai S, Burnette B, Stack M, Oshima G, Khan S, Huang X, Posner MC, Weichselbaum RR, Khodarev NN (2015). Oncogenic CXCL10 signalling drives metastasis development and poor clinical outcome. Br J Cancer.

[73] Wu Q, Dhir R, Wells A (2012). Altered CXCR3 isoform expression regulates prostate cancer cell migration and invasion. Mol Cancer.

[74] Furuya M, Yoneyama T, Miyagi E, Tanaka R, Nagahama K, Miyagi Y, Nagashima Y, Hirahara F, Inayama Y, Aoki I (2011). Differential expression patterns of CXCR3 variants and corresponding CXC chemokines in clear cell ovarian cancers and endometriosis. Gynecol Oncol.

[75] Ehlert JE, Addison CA, Burdick MD, Kunkel SL, Strieter RM (2004). Identification and partial characterization of a variant of human CXCR3 generated by posttranscriptional exon skipping. J Immunol.

[76] Jiskra J, Antosova M, Kratky J, Vitkova H, Limanova Z, Mareckova H, Potlukova E (2016). CXCR3, CCR5, and CRTH2 chemokine receptor expression in lymphocytes infiltrating thyroid nodules with coincident hashimoto's thyroiditis obtained by fine needle aspiration biopsy. J Immunol Res.

[77] Lee JH, Kim Y, Choi JW, Kim YS (2013). The association between papillary thyroid carcinoma and histologically proven hashimoto's thyroiditis: a meta-analysis. Eur J Endocrinol.

[78] Beider K, Nagler A, Wald O, Franitza S, Dagan-Berger M, Wald H, Giladi H, Brocke S, Hanna J, Mandelboim O, Darash-Yahana M, Galun E, Peled A (2003). Involvement of CXCR4 and IL-2 in the homing and retention of human NK and NK T cells to the bone marrow and spleen of nod/scid mice. Blood.

[79] Moser B, Loetscher P (2001). Lymphocyte traffic control by chemokines. Nat Immunol.

[80] Billottet C, Quemener C, Bikfalvi A (2013). CXCR3, a double-edged sword in tumor progression and angiogenesis. Biochim Biophys Acta.

[81] Loetscher M, Gerber B, Loetscher P, Jones SA, Piali L, Clark-Lewis I, Baggiolini M, Moser B (1996). Chemokine receptor specific for IP10 and mig: structure, function, and expression in activated t-lymphocytes. J Exp Med.

[82] Loetscher M, Loetscher P, Brass N, Meese E, Moser B (1998). Lymphocyte-specific chemokine receptor CXCR3: regulation, chemokine binding and gene localization. Eur J Immunol.

[83] Datta D, Contreras AG, Grimm M, Waaga-Gasser AM, Briscoe DM, Pal S (2008). Calcineurin inhibitors modulate CXCR3 splice variant expression and mediate renal cancer progression. J Am Soc Nephrol.

[84] Zipin-Roitman A, Meshel T, Sagi-Assif O, Shalmon B, Avivi C, Pfeffer RM, Witz IP, Ben-Baruch A (2007). CXCL10 promotes invasion-related properties in human colorectal carcinoma cells. Cancer Res.

[85] Gacci M, Serni S, Lapini A, Vittori G, Alessandrini M, Nesi G, Palli D, Carini M (2009). CXCR3-B expression correlates with tumor necrosis extension in renal cell carcinoma. J Urol.

[86] Hu M, Li K, Maskey N, Xu Z, Yu F, Peng C, Li Y, Yang G (2015). Overexpression of the chemokine receptor CXCR3 and its correlation with favorable prognosis in gastric cancer. Hum Pathol.

[87] Evdokimova V, Tognon C, Ng T, Sorensen PH (2009). Reduced proliferation and enhanced migration: two sides of the same coin? molecular mechanisms of metastatic progression by YB-1. Cell Cycle.

[88] Romagnani P, Beltrame C, Annunziato F, Lasagni L, Luconi M, Galli G, Cosmi L, Maggi E, Salvadori M, Pupilli C, Serio M (1999). Role for interactions between IP-10/Mig and CXCR3 in proliferative glomerulonephritis. J Am Soc Nephrol.

[89] Romagnani P, Lazzeri E, Lasagni L, Mavilia C, Beltrame C, Francalanci M, Rotondi M, Annunziato F, Maurenzig L, Cosmi L, Galli G, Salvadori M, Maggi E (2002). IP-10 and Mig production by glomerular cells in human proliferative glomerulonephritis and regulation by nitric oxide. J Am Soc Nephrol.

[90] Sethi G, Sung B, Aggarwal BB (2008). TNF: a master switch for inflammation to cancer. Front Biosci.

[91] Balkwill F (2009). Tumour necrosis factor and cancer. Nat Rev Cancer.

[92] Sun KH, Sun GH, Wu YC, Ko BJ, Hsu HT, Wu ST (2016). TNF-alpha augments CXCR2 and CXCR3 to promote progression of renal cell carcinoma. J Cell Mol Med.

[93] Antonelli A, Ferrari SM, Corrado A, Di Domenicantonio A, Fallahi P (2015). Autoimmune thyroid disorders. Autoimmun Rev.

[94] Antonelli A, Ferrari SM, Giuggioli D, Ferrannini E, Ferri C, Fallahi P (2014). Chemokine (C-X-C motif) ligand (CXCL)10 in autoimmune diseases. Autoimmun Rev.

[95] Melillo RM, Guarino V, Avilla E, Galdiero MR, Liotti F, Prevete N, Rossi FW, Basolo F, Ugolini C, de Paulis A, Santoro M, Marone G (2010). Mast cells have a protumorigenic role in human thyroid cancer. Oncogene.

[96] Khazaie K, Blatner NR, Khan MW, Gounari F, Gounaris E, Dennis K, Bonertz A, Tsai FN, Strouch MJ, Cheon E, Phillips JD, Beckhove P, Bentrem DJ (2011). The significant role of mast cells in cancer. Cancer Metastasis Rev.

[97] Hussain SP, Harris CC (2007). Inflammation and cancer: an ancient link with novel potentials. Int J Cancer.

[98] Maeda H, Akaike T (1998). Nitric oxide and oxygen radicals in infection, inflammation, and cancer. Biochemistry.

[99] Lleo A, Zhang W, Zhao M, Tan Y, Bernuzzi F, Zhu B, Liu Q, Tan Q, Malinverno F, Valenti L, Jiang T, Tan L, Liao W (2015). DNA methylation profiling of the X chromosome reveals an aberrant demethylation on CXCR3 promoter in primary biliary cirrhosis. Clin Epigenetics.

[100] Sampath Kumar DS, Wells A (2013). Abstract 2973: CXCR3 epigenome switches splice variants in prostate cancer. Cancer Res.

[101] Lemoine NR, Mayall ES, Jones T, Sheer D, McDermid S, Kendall-Taylor P, Wynford-Thomas D (1989). Characterisation of human thyroid epithelial cells immortalised *in vitro* by simian virus 40 DNA transfection. Br J Cancer.

[102] Schweppe RE, Klopper JP, Korch C, Pugazhenthi U, Benezra M, Knauf JA, Fagin JA, Marlow LA, Copland JA, Smallridge RC, Haugen BR (2008). Deoxyribonucleic acid profiling analysis of 40 human thyroid cancer cell lines reveals cross-contamination resulting in cell line redundancy and misidentification. J Clin Endocrinol Metab.

[103] Pfaffl MW (2001). A new mathematical model for relative quantification in real-time RT-PCR. Nucleic Acids Res.

[104] Zhou SF, Ma J, Qu HT, Liu ZT, He WD, Wang JD, Dou AX, Zhang N, Liu JL, Guo CS, Shi Y, Hou M, Peng J (2013). Characterization of th1- and th2-associated chemokine receptor expression in spleens of patients with immune thrombocytopenia. J Clin Immunol.

[105] Latini FR, Hemerly JP, Oler G, Riggins GJ, Cerutti JM (2008). Re-expression of abi3-binding protein suppresses thyroid tumor growth by promoting senescence and inhibiting invasion. Endocr Relat Cancer.

