# Supplementary material for: Differential expression profile of CXCR3 splicing variants is associated with thyroid neoplasia. Potential role in papillary thyroid carcinoma oncogenesis?
Source: Oncotarget. 2017 Dec 20;9(2):2445–67. doi: 10.18632/oncotarget.23502 (PMC5788652; doi:10.18632/oncotarget.23502)
Supplement: Supplementary file 1 [file oncotarget-09-2445-s001.pdf]

Differential expression profile of CXCR3 splicing variants is associated with thyroid neoplasia. Potential role in papillary thyroid carcinoma oncogenesis?

SUPPLEMENTARY MATERIALS

RNA extraction and cDNA synthesis

Total RNA was isolated using the RNeasy Plus Mini kit (Qiagen CA) according to the manufacturer's instructions and stored at -80°C. RNA concentration was determined by the Picocube spectrophotometer (Picodrop). Samples with concentration <88 ηg/µl were excluded in this study as the RT reaction was adjusted for 1000 ηg of RNA. Complementary DNA (cDNA) synthesis was performed using the ImProm-II Reverse Transcription System (Promega) according to the manufacturer's instructions.

Real-Time PCR (qPCR) reaction and qPCR analyses

Reactions were initiated with a 10 min. denaturation step at 95˚C, followed by 45 cycles of denaturation at 95˚C for 20 sec., 20 sec. of annealing at 60˚C, and 20 sec. of extension at 72˚C. Fluorescence was measured at the end of each cycle. Amplicons were subjected to melting curve analysis by increasing the temperature from 72°C to 95°C at an increment of 1°C per second. Ct (cycle threshold) values over 35 and deficient melting curves were not considered.

The RFC for over-expressed genes was calculated considering the difference between their Pfaffl's ratio value and 1 (the "control value") while under-expressed genes considered the negative difference between 1 and the multiplicative inverse value of their Pfaffl's ratio.

CXCR3A and CXCR3B standard curves and copy number quantification

Plasmid with complete coding sequence for human CXCR3A, CXCR3B and ACTB were purchased from GenScript (Piscataway, NJ). Concentration of CXCR3 variants and ACTB was determined by performing standard curves with ten-fold serial dilutions of each plasmid ranging from 10 ηg/µl to 10 ag/µl. Each point was assayed in triplicate. Copy number (cn) quantification for CXCR3 splicing variants was calculated by interpolating Ct values of CXCR3A and CXCR3B into their respective plasmids standard curve. Total µg of CXCR3A, CXCR3B and ACTB were converted to cn values following this equation:

$$\text{Copy Number} = \frac{\text{ds DNA (mg)}*6{,}022*10^{23}}{\#\,\text{pb}*10^{3}*660}$$

The relative CXCR3A or CXCR3B mRNA levels related to CXCR3 were displayed as CXCR3A/CXCR3 and CXCR3B/CXCR3 cn ratios.

Immunohistochemistry

Five µm paraffin-embedded sections were de-waxed and rehydrated in TBS buffer. Endogenous peroxidase was blocked with H_2O_2 1% in methanol for 20 minutes at room temperature. Sections were incubated with blocking solution (DakoCytomation, X0909) for 1 hour at room temperature followed by overnight incubation with primary monoclonal (mAb) anti–human CXCR3 antibody (Abcam, ab64714, lot: GR46157-1) diluted 1:250 or with a polyclonal anti-human CXCL10 antibody (R&D Systems, AF-266-NA, lot: ABR050821) diluted 1:50. Sections were washed 3 times for 5 minutes with TBS 0.5% Tween, incubated with a secondary biotinylated anti-mouse antibody (Vector, BA-2000) for one hour at 4°C followed by 3 times for 5 min. washes with TBS Tween 0.5%. Immunoreactivity was revealed with substrate–chromogen solution ImmPACT DAB (Vector, SK-4105). Sections were stained with hematoxylin (Merck, 1.05174.0500).

Western blot

Primary mouse monoclonal antibody for total CXCR3 (1:1000; Abcam, ab64714, lot: GR46157-1), mouse pAb for CXCR3 variants (1:1000; R&D Systems, MAB160, lot: AOU1011121) and mouse mAb for α-tubulin (1:1000; SIGMA, T9026, lot: 092M4792) were used. Thyroid specimens in protein lysis buffer, (Tris-HCl 20 mM pH 7.6, EDTA 1 mM, EGTA 0.5 mM, sucrose 250 mM, triton X-100 1%, $Na_4O_7P_2$ 10 mM, NaF 50 mM, $NaVO_3$ 1 mM) supplemented with protease inhibitors (Sigma) were disrupted by intermittent homogenization on ice. Cultured cells were resuspended in lysis buffer and incubated 30 min. on ice with intermittent vortexing. Lysates were centrifuged and supernatants were collected. Protein concentration was determined by micro BCA method (Bio-Rad). Loading samples were prepared by mixing 60 µg of total proteins with Laemmli's buffer

(62.5 ηM Tris-HCl pH 6.8, 10% v/v glycerol, 2.5% w/v SDS, 3% v/v 2-mercaptoethanol, 5% w/v bromophenol blue). Proteins were separated by 10% SDS-PAGE and electro-transferred into a nitrocellulose membrane. After 1 h. incubation in 5% non-fat milk-TBS (10 mM Tris-HCl pH 8.0, 150 mM NaCl) the membrane was incubated overnight at 4°C with primary antibody. After three washes for 5 min. in TBS-Tween 0.1%, the membrane was incubated with HRP-conjugated secondary antibody. Western lightning ® Plus-ECL (Perkin Elmer) was used for protein detection. Membranes were exposed to autoradiography films and manually developed. Protein signal intensity was evaluated by densitometry analysis by using the ImageJ version 1.46p software (National Institutes of Health, USA). Densitometric values for target bands were normalized by the reference gene densitometry value (α-tubulin).

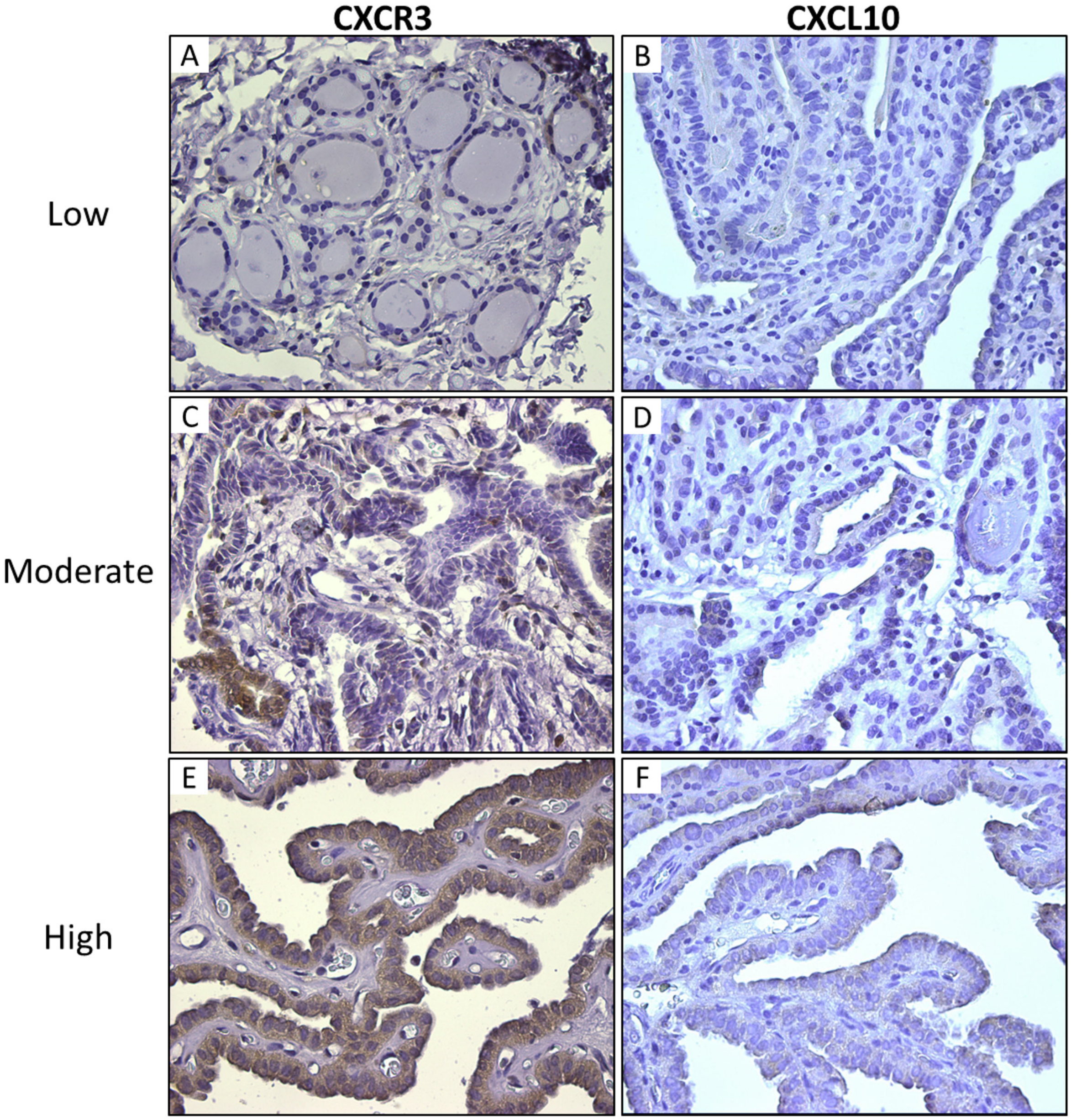

Supplementary Figure 1: Representative immunohistochemistry images of CXCR3 and CXCL10 percentage of positive samples (low, moderate and high) in thyroid tissues. (A-B) Low staining (0-33%). **(C-D)** Moderate staining (34-66%). **(E-F)** high staining (67-100%). A, PTC-CLT sample. B-F, PTC samples. A-F, Magnification, ×400. A, C and E: CXCR3 staining. B, D and F: CXCL10 staining.

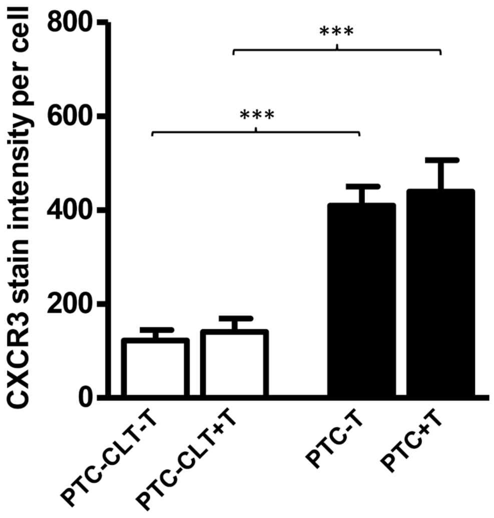

Supplementary Figure 2: Expression of total CXCR3 receptor in PTC tissues with thyroiditis (PTC+T) and without thyroiditis (PTC-T). Quantitative analysis of average staining intensity for CXCR3 expression by immunohistochemistry was performed in PTC tissues and compared to their respective (PTC-CLT). PTC samples were additionally categorized for the presence of thyroiditis. Values plotted represent the relative expression levels of CXCR3 receptor in PTC (black bar) and in PTC-CLT (white bar) with thyroiditis (+T, n=10) or without thyroiditis (-T, n=20) normalized by total cell number analyzed in each slide. Data represent mean ± s.e.m. Statistical analysis was performed using an unpaired two-tailed t-test. ($^{*}p<0.05$, $^{**}p<0.01$, $^{***}p<0.001$).

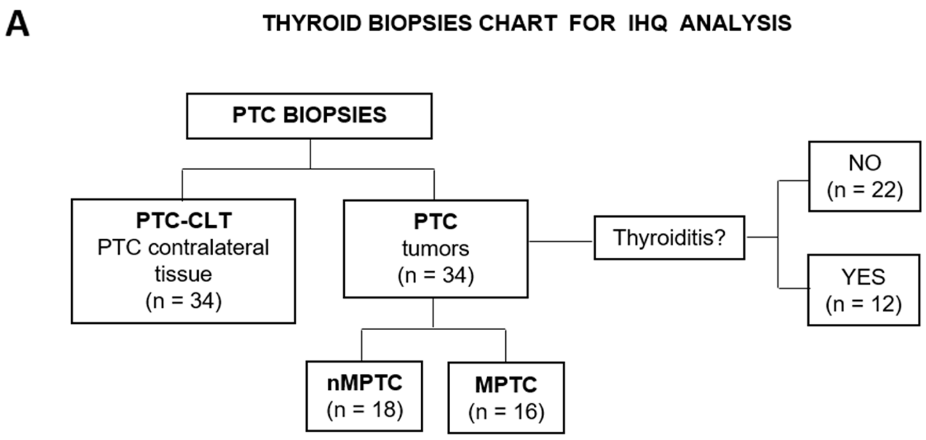

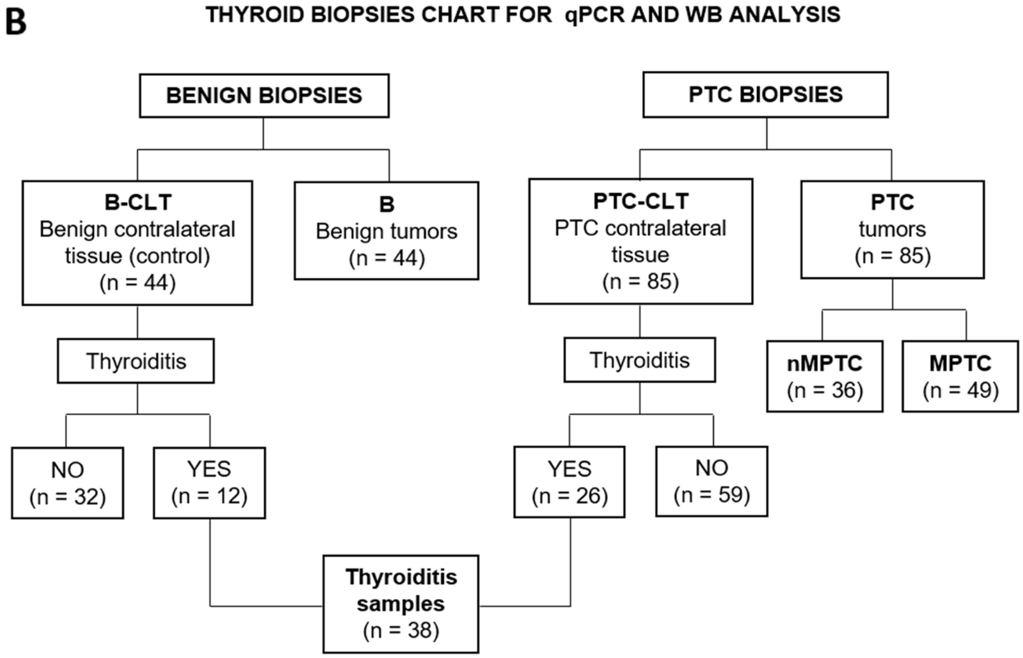

Supplementary Figure 3: Thyroid Biopsy charts for IHQ **(A)**, qPCR and WB **(B)** analysis. Benign contralateral tissue (B-CLT) was considered as the gold standard for thyroid control tissue. Benign tumors (B) were considered as neoplastic and non-malignant tissue; PTC contralateral tissue (PTC-CLT) were considered as non-neoplastic and non-malignant tissue; papillary thyroid cancer tumors (PTC) were considered as neoplastic and malignant tissue. PTC tumors were categorized in non-metastatic PTC (nMPTC) and metastatic PTC (MPTC) tissues. B-CLT and PTC-CLT with moderate or severe reported thyroiditis by histopathological diagnosis were considered as thyroiditis samples.

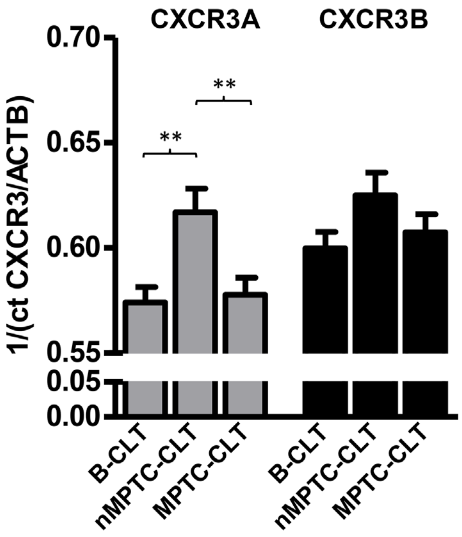

Supplementary Figure 4: Differential expression profile of CXCR3A and CXCR3B in contralateral tissues from benign and cancer thyroid tissues. mRNA expression levels of target genes were determined by qPCR in contralateral tissues from benign, non-metastatic PTC and metastatic PTC tissues named as (B-CLT), (nMPTC-CLT) and (MPTC-CLT) respectively. The graph shows the reciprocal value for CXCR3A/ACTB and CXCR3B/ACTB ct ratio in 30 B-CLT, 40 nMPTC-CLT and in 35 MPTC-CLT. Data are shown as mean ± s.e.m. Statistical significance for reciprocal values (1/CXCR3A or CXCR3B/ACTB) was analyzed by unpaired two-tailed t-test. (*p<0.05, **p<0.01, ***p<0.001).

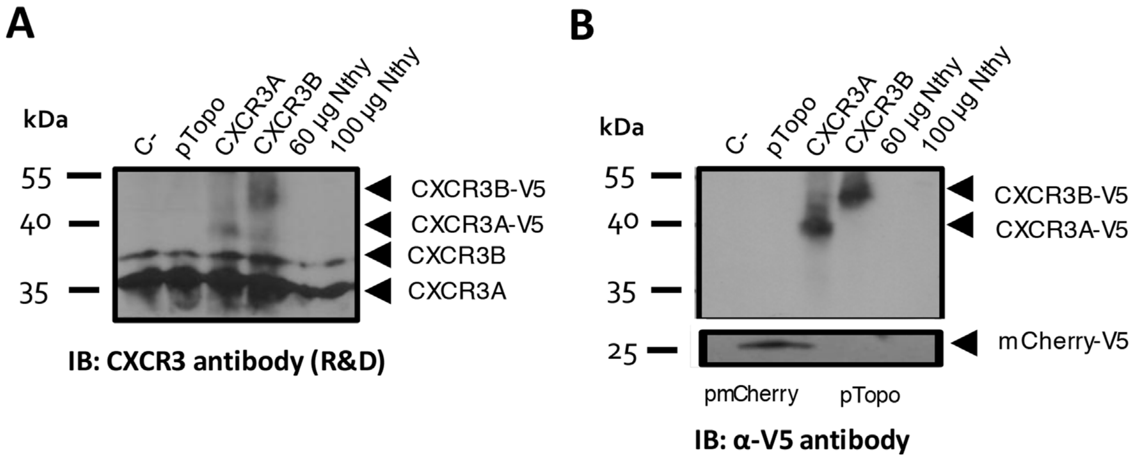

Supplementary Figure 5: CXCR3 antibody validation. The CXCR3 antibody from R&D Systems, MAB160, used to detect the CXCR3A and CXCR3B isoforms in thyroid tissues by western blot analysis was validated by a molecular approach that combined T7 RNA polymerase based *in vitro* transcription followed by *in vitro* translation in rabbit reticulocyte lysate (RRL, Promega). DNA plasmids, harboring the T7 RNA polymerase promoter, encoding for the V5-tagged CXCR3A-V5 or CXCR3B-V5 isoforms linearized by PmeI digestion were used as templates in a T7 polymerase based *in vitro* transcription reaction. The generated RNA was used to program RRL and an *in vitro* translation was performed. **(A)** Translation products were analyzed by western blot analysis using the CXCR3 R&D antibody or **(B)** an anti-V5 monoclonal antibody (upper panel). Positive results were considered when the fused CXCR3A-V5 and CXCR3-V5 isoforms generated a V5 signal that overlapped with the CXCR3 labeling with an expected molecular weight for each CXCR3 monomer. The western blot analysis showed a V5 signal that overlapped with the R&D labeling of 44.5 kDa and 50.4 kDa for CXCR3A-V5 and CXCR3B-v5, respectively. This validation test demonstrates that the CXCR3 R&D antibody effectively recognizes the CXCR3A and CXCR3B splicing variants. C-; Total reticulocytes without RNA transcription. pmCherry-V5: positive control for V5 monoclonal antibody. 20 µL of *in vitro* translation reaction were loaded from pTopo (empty vector), CXCR3A and CXCR3B.
